# Big data analytics and sentiment analysis for health misinformation detection: a systematic literature review of influencing factors, implementation challenges and a data-driven adoption framework

**DOI:** 10.3389/fdata.2026.1948398

**Published:** 2026-10-05

**Authors:** Khurram Shahzad, Asfa Muhammed Din Javeed, Shakil Ahmad, Mujahid Latif, Kashaf Saleem

**Affiliations:** 1 Government College University, Lahore, Pakistan; 2 Alfaisal University, Riyadh, Saudi Arabia; 3 Prince Sultan University, Riyadh, Saudi Arabia; 4 University of Salford, Salford, United Kingdom

**Keywords:** adoption challenges, big data analytics, health misinformation, influencing factors, sentiment analysis, SLR

## Abstract

The study aimed to identify the factors influencing big data analytics (BDA) and sentiment analysis (SA) for health misinformation detection and associated challenges for the adoption of BDA and SA across health ecosystems. The systematic literature review (SLR) methodology was applied for addressing the study's objectives. Thirty most relevant seminal studies (peer-reviewed articles and conference proceedings) were retrieved from 11 digital platforms to conduct the SLR. Results of the study showed that advanced deep learning architectures, feature engineering, sentiment intelligence, real-time analytics, and human-centered verification were key factors that influenced big data analytics and sentiment analysis based health misinformation detection. It was also identified that annotation limitations, scalability constraints, domain adaptation challenges, ethical concerns, and sustainability challenges negatively impacted the adoption of big data and sentiment driven health misinformation detection systems. The study has developed a data-driven framework to effectively adopt and sustain big data analytics and sentiment analysis to detect health misinformation.

**Systematic review registration:** https://osf.io/wjrm6.

## 1 Introduction

Misinformation is a dominating challenge in the ever-changing digital revolutionary world. In the current age, excessive amount of generated content on digital media has caused concerns related to the accuracy and genuineness of the information being disseminated (Arias et al., 2022; Di Sotto and Viviani, 2022). The creators of misinformation related to health apply different techniques for the promotion of their content to arouse people's sentiments. It has also encouraged the use of sentiment analysis (SA) to capture emotional polarity, attitudes, and opinions expressed in health-related social-media content. Such sentiment-derived information can provide an auxiliary signal for misinformation detection by helping characterize communication patterns and potentially manipulative charged content; however, sentiment analysis alone cannot determine whether a health claim is factually accurate, credible, or misleading (Alonso et al., 2021).

Conceptually, sentiment analysis and misinformation detection represent related but distinct analytical tasks. Sentiment analysis primarily identifies and classifies affective polarity, emotions, attitudes, and opinions expressed in textual and social-media content, whereas misinformation detection seeks to determine whether a claim and information item is inaccurate, misleading, deceptive, and unsupported by available evidence. Consequently, a positive, negative, and neutral sentiment does not establish the truthfulness of a health claim. For example, both factual and misleading health information may be expressed with positive and negative sentiment. Within the context of this review, sentiment analysis is therefore treated as an auxiliary feature that may complement semantic, linguistic, behavioral, temporal, network, source-credibility, and evidence-based approaches to misinformation detection. The actual determination of misinformation requires broader contextual and verification mechanisms rather than sentiment polarity alone.

Digital misinformation on social networks misguides people and negatively affects their sentiments about health related matters (Morita et al., 2023). Authenticity of online health information is difficult due to determine due to the unavailability of quality data (Villanueva-Miranda et al., 2025). Lack of expert system for analyzing huge volumes of social media data, health officials might not detect health misinformation effectively and efficiently (Arora et al., 2026; Cavus et al., 2025; George and Baskar, 2024; Hussain et al., 2023).

The vast amount of online information has brought a paradigm shift in big data analytics (BDA) innovations as it assists to take evidence-based decisions in healthcare sector (Cano-Marin et al., 2023). BDA and natural language processing transformers support the extraction and analysis of sentiment, semantic, linguistic, and contextual signals that can contribute to health misinformation detection (Sharma et al., 2025). Deep learning algorithms and NLP feature extraction allow for nuanced sentiment and emotional tone analysis, enhancing detection frameworks designed to identify deceptive or fabricated user-generated content (Wani et al., 2024). Machine learning techniques are used to develop sentiment models that extract affective and opinion-related features which may contribute to broader misinformation detection systems (Kabir, 2022). Through big data analytics and deep learning algorithms, sentiment-related features can be incorporated into broader frameworks for analyzing and detecting health misinformation (Zamir et al., 2024). BDA can support health professionals in analyzing large-scale health information environments, while sentiment analysis can provide complementary signals within broader misinformation detection and verification processes (Cynthia et al., 2026; Feng et al., 2025; Liu et al., 2019).

Advanced computational techniques can improve sentiment analysis and other contextual representations that contribute to the identification and analysis of health misinformation (Taha, 2025). The integration of big data analytics (BDA) with deep neural network (DNN) proves fruitful in detecting health misinformation from vast amounts of online datasets (Babar et al., 2024). Natural language processing innovations support in reliable sentiment analysis for public health safety through digital informational content analysis (Marques et al., 2022). BDA innovations assist in filtering online datasets for detecting health misinformation to facilitate different stakeholders in order to sustain a healthy and smart city (Azzaoui et al., 2021). Artificial intelligence, neural networks, and fact-checking resources can jointly support misinformation detection, while sentiment analysis may provide additional information about the emotional and attitudinal characteristics of potentially misleading content (Shahzad et al., 2022). Lexicon-based scoring algorithms can characterize sentiment-related signals within health information, which may subsequently be combined with other features for misinformation analysis (Balshetwar et al., 2023; Chatta et al., 2026).

Certain challenges are associated with sentiment analysis on social media data. To accurately identify sentiment of slangs, short texts, emojis, contexts, and linguistic differences from various social media platforms is a prominent challenge (Kansara and Adhvaryu, 2024). The continuous spread of harmful data by spammers on social media causes challenges for healthcare stakeholders to detect online health misinformation (Kansara and Adhvaryu, 2022). Campaigns by different fake content generators on social media to receive public attraction are a critical barrier to the credibility of health related information (Alarabid et al., 2026). Unclear policies about fake online health data lead to serious problems for the incorporation of big data analytics sentiment-driven misinformation detection systems in healthcare information environments (Hussain et al., 2023). Algorithm biases create problems for an efficient and sustainable adoption of big data analytics and sentiment analysis to detect health misinformation (Ahmed and Ahmed, 2023; Malek et al., 2026; Wreyford et al., 2026).

### 1.1 Statement of the problem

In the current age of rapidly changing technological innovations, health misinformation posted through social media forums, digital communication platforms, and online news portals is a critical challenge. Sentiment analysis (SA) has shown considerable potential for identifying users' emotions, opinions, and attitudes toward health-related information. However, sentiment characteristics alone cannot establish the factuality of health claims. Although BDA and SA can contribute to online health misinformation detection, their effective use requires the integration of sentiment-derived signals with contextual semantics, linguistic characteristics, source credibility, behavioral patterns, temporal information, and evidence-based verification. Therefore, an important research challenge is to understand how BDA and sentiment-related analytical signals can be integrated into broader misinformation detection ecosystems while avoiding the assumption that emotional polarity itself represents evidence of misinformation. The increasing volume, velocity, variety, and veracity of health-related data created on social networking sites have caused challenges to detect health misinformation for making informed decisions related to healthcare. In this context, big data analytics (BDA) is an effective approach to process and analyze heterogeneous datasets in real time, and sentiment analysis (SA) has shown positive prospects in identifying users' emotions, opinions, and attitudes toward health-related information. Although BDA and SA have a potential to detect online health misinformation notwithstanding the existing body of knowledge remains dispersed across diverse disciplines, application domains, and methodologies.

Previous investigations have positively considered the effectiveness of big data analytics, and sentiment analysis approaches in identifying, evaluating, and predicting health misinformation posted through digital platforms. In past, various researchers proposed data science-based approaches and analytical frameworks for detecting health misinformation on social media (Di Sotto and Viviani, 2022; Morita et al., 2023; Zakir Hussain et al., 2023). Similarly, studies have indicated the usefulness of sentiment analysis to examine public perspectives, sentimental responses, and behavioral intentions associated with health misinformation, fake news, and public health crises (Kabir, 2022; Marques et al., 2022; Villanueva-Miranda et al., 2025). Recent investigations have further demonstrated the incorporation of deep learning models, machine learning, transformer architectures, cloud-based systems, and large language models (LLM) to enhance the scalability of misinformation detection systems (Alarabid et al., 2026; Babar et al., 2024; Cavus et al., 2025; Feng et al., 2025; Malek et al., 2026). Although several reviews, surveys, and scientometric studies have examined fake news detection, sentiment analysis, and big data applications independently (Cano-Marin et al., 2023; Kansara and Adhvaryu, 2024; Shahzad et al., 2022), however a comprehensive systematic literature review (SLR) based study focusing on the factors influencing big data analytics and sentiment analysis-based approaches for health misinformation detection has not been conducted yet. It is a pertinent gap in existing body of knowledge that needs to be bridged.

The current study has conducted an in-depth SLR of peer-reviewed journal articles and conference proceedings (*n* = 30) to consolidate the existing evidence on the factors influencing the adoption of big data analytics and sentiment analysis for health misinformation detection. The study has addressed the need to identify the factors influencing big data analytics and sentiment analysis-based approaches for health misinformation detection, and revealed the challenges associated with the implementation of big data analytics and sentiment-driven misinformation detection systems in healthcare information environments. Based on the study's findings, a data-driven framework has been developed for the sustainable adoption of these technologies for detecting online health misinformation. The study has added a substantial amount of literature in existing body of knowledge by identifying the factors influencing the adoption of BDA and SA for health misinformation detection. It has provided practical implications for policy makers and relevant stakeholders to efficiently adopt and sustain BDA and SA for public health care.

### 1.2 Objectives of the study

The objectives of the study are as follows:

- To identify the factors influencing big data analytics and sentiment analysis-derived signals within health misinformation detection approaches.
- To reveal the challenges associated with the implementation of big data and sentiment-driven misinformation detection systems in healthcare information environments.

### 1.3 Research questions of the study

The study's research questions are as follows:

- Which factors influence big data analytics and sentiment analysis-derived signals within health misinformation detection approaches?
- Which challenges are associated with the implementation of big data and sentiment-driven misinformation detection systems in healthcare information environments?
- What is an effective framework to adopt big data analytics and sentiment analysis for health misinformation detection on social media?

## 2 Materials and methods

### 2.1 SLR methodology

To address the study's research questions, systematic literature review (SLR) methodology was employed. An SLR is applied using reproducible, transparent, and structured techniques through pre-developed standards. These standards are regarded as protocols that offer insights for all phases of the systematic review based studies to maintain rigor, accuracy, efficiency, and authenticity. These protocols increase reliability, credibility, and validity of research and provide an explicit roadmap for future researchers to conduct similar studies using other dimensions (Ali et al., 2026; Shahzad et al., 2024, 2026; Shahzad and Tariq, 2025).

### 2.2 Usage of PRISMA

The current study was executed through the Preferred Reporting Items for Systematic Review and Meta-Analyses (PRISMA 2020) guidelines. The review applied the PRISMA framework to ensure methodological transparency, reproducibility, and uniformity. A four-phase flow diagram was displayed to manifest the entire study's selection procedure. The SLR protocol was registered in the Open Science Framework (OSF) and is accessible through: osf.io/wjrm6. Furthermore, the PRISMA 2020 checklist was followed that is shown through Table 1. Each reporting is aligned systematically with the corresponding sections of the review. The required core studies (*n* = 30) were identified through the criteria of inclusion and exclusion for the final selection to conduct the systematic review on the focused topic.

**Table 1:** PRISMA 2020 checklist.

Section and topic	Item #	Checklist item	Location where item is reported
Title			
Title	1	Identify the report as a systematic review	Title (“systematic literature review”)
Abstract			
Abstract	2	See the PRISMA 2020 for Abstracts checklist	Abstract section
Introduction			
Rationale	3	Describe the rationale for the review in the context of existing knowledge	Introduction; Statement of the Problem
Objectives	4	Provide an explicit statement of the objective(s) or question(s) the review addresses	Objectives of the study; research questions of the study
Methods			
Eligibility criteria	5	Specify the inclusion and exclusion criteria for the review and how studies were grouped for the syntheses	Selection; Table 3 (search results from databases and tools)
Information sources	6	Specify all databases, registers, websites, organizations, reference lists and other sources searched or consulted to identify studies. Specify the date when each source was last searched or consulted	Selection; Table 4 (search strategies); Table 3 (search queries and filters)
Search strategy	7	Present the full search strategies for all databases, registers and websites, including any filters and limits used	Table 3
Selection process	8	Specify the methods used to decide whether a study met the inclusion criteria of the review, including how many reviewers screened each record and each report retrieved, whether they worked independently, and if applicable, details of automation tools used in the process	Extraction; Table 6 (CASP checklist); quality mapping scores (Table 7); structured data extraction form (Table 5 description)
Data collection process	9	Specify the methods used to collect data from reports, including how many reviewers collected data from each report, whether they worked independently, any processes for obtaining or confirming data from study investigators, and if applicable, details of automation tools used in the process	Extraction; Table 6 (CASP checklist); quality mapping scores (Table 7)
Data items	10a	List and define all outcomes for which data were sought. Specify whether all results that were compatible with each outcome domain in each study were sought (e.g., for all measures, time points, analyses), and if not, the methods used to decide which results to collect	Table 5
	10b	List and define all other variables for which data were sought (e.g., participant and intervention characteristics, funding sources). Describe any assumptions made about any missing or unclear information	Extraction; Table 6 (CASP checklist); quality mapping scores (Table 7)
Study risk of bias assessment	11	Specify the methods used to assess risk of bias in the included studies, including details of the tool(s) used, how many reviewers assessed each study and whether they worked independently, and if applicable, details of automation tools used in the process	Extraction; Table 6 (CASP checklist); quality mapping scores (Table 7)
Effect measures	12	Specify for each outcome the effect measure(s) (e.g., risk ratio, mean difference) used in the synthesis or presentation of results	Execution (thematic analysis); Table 5
Synthesis methods	13a	Describe the processes used to decide which studies were eligible for each synthesis [e.g., tabulating the study intervention characteristics and comparing against the planned groups for each synthesis (item #5)]	Extraction; execution (thematic analysis)
	13b	Describe any methods required to prepare the data for presentation or synthesis, such as handling of missing summary statistics, or data conversions	Table 5 and Figures 2–5
	13c	Describe any methods used to tabulate or visually display results of individual studies and syntheses	Execution (thematic analysis)
	13d	Describe any methods used to synthesize results and provide a rationale for the choice(s). If meta-analysis was performed, describe the model(s), method(s) to identify the presence and extent of statistical heterogeneity, and software package(s) used	Section 2.9 (execution and thematic synthesis); structured thematic synthesis was used to code, group, compare, and interpret findings across the included studies
	13e	Describe any methods used to explore possible causes of heterogeneity among study results (e.g., subgroup analysis, meta-regression)	No quantitative effect estimates were synthesized. Methodological, geographical, contextual, and design-related variation across studies was considered during thematic comparison and interpretation
	13f	Describe any sensitivity analyses conducted to assess robustness of the synthesized results	Not applicable; no quantitative sensitivity analysis was conducted
Reporting bias assessment	14	Describe any methods used to assess risk of bias due to missing results in a synthesis (arising from reporting biases)	Not formally assessed because no quantitative synthesis was conducted
Certainty assessment	15	Describe any methods used to assess certainty (or confidence) in the body of evidence for an outcome	No formal certainty of evidence framework was applied. Instead, the methodological quality of individual studies was assessed using the CASP-informed framework, and quality scores were considered when interpreting the synthesized evidence
Results			
Study selection	16a	Describe the results of the search and selection process, from the number of records identified in the search to the number of studies included in the review, ideally using a flow diagram	Section 2.5 (selection); Figure 1 (PRISMA flow diagram)
	16b	Cite studies that might appear to meet the inclusion criteria, but which were excluded, and explain why they were excluded	Table 6 (CASP checklist for quality appraisal)
Study characteristics	17	Cite each included study and present its characteristics	Table 5 (structured data-extraction form); results-characteristics of included studies
Risk of bias in studies	18	Present assessments of risk of bias for each included study	Results; Table 6 (CASP checklist for quality appraisal)
Results of individual studies	19	For all outcomes, present, for each study: (a) summary statistics for each group (where appropriate), and (b) an effect estimate and its precision (e.g., confidence/credible interval), ideally using structured tables or plots	Not applicable; no quantitative outcomes, effect estimates, or statistical synthesis were reported. Findings were synthesized thematically, while methodological quality/risk of bias was assessed separately using the CASP-informed quality appraisal framework (Table 6)
Results of syntheses	20a	For each synthesis, briefly summarize the characteristics and risk of bias among contributing studies	Results; Table 6 (CASP checklist for quality appraisal); thematic synthesis; Table 7 (quality mapping score)
	20b	Present results of all statistical syntheses conducted. If meta-analysis was done, present for each the summary estimate and its precision (e.g., confidence/credible interval) and measures of statistical heterogeneity. If comparing groups, describe the direction of the effect	Not applicable; no statistical synthesis was conducted
	20c	Present results of all investigations of possible causes of heterogeneity among study results	Not applicable; no statistical heterogeneity was calculated. Methodological and contextual variation was considered narratively during thematic synthesis
	20d	Present results of all sensitivity analyses conducted to assess the robustness of the synthesized results	Not applicable; no quantitative sensitivity analyses were conducted
Reporting biases	21	Present assessments of risk of bias due to missing results (arising from reporting biases) for each synthesis assessed	No formal certainty of evidence assessment was conducted. The methodological quality of individual studies was assessed using the CASP-informed appraisal framework and considered during interpretation
Certainty of evidence	22	Present assessments of certainty (or confidence) in the body of evidence for each outcome assessed	Limitations and future research directions
Discussion			
Discussion	23a	Provide a general interpretation of the results in the context of other evidence	Discussion; framework; implications; and conclusion
	23b	Discuss any limitations of the evidence included in the review	Limitations and future research directions sections
	23c	Discuss any limitations of the review processes used	Limitations and future research directions; methodological limitations of the search, selection, language restriction, database coverage, evidence heterogeneity, and other relevant limitations are discussed
	23d	Discuss implications of the results for practice, policy, and future research	Implications; limitations and future research directions; conclusion
Other information			
Registration and protocol	24a	Provide registration information for the review, including register name and registration number, or state that the review was not registered	The review was registered in open science framework
	24b	Indicate where the review protocol can be accessed, or state that a protocol was not prepared	The review protocol can be accessed via: osf.io/wjrm6
	24c	Describe and explain any amendments to information provided at registration or in the protocol	No amendments to information provided at registration are done
Support	25	Describe sources of financial or non-financial support for the review, and the role of the funders or sponsors in the review	No financial or non-financial support was received for conducting this review
Competing interests	26	Declare any competing interests of review authors	The authors declare no competing interests
Availability of data, code and other materials	27	Report which of the following are publicly available and where they can be found: template data collection forms; data extracted from included studies; data used for all analyses; analytic code; any other materials used in the review	The study characteristics, structured data-extraction information, and study-level methodological quality assessments are reported in the manuscript tables. No separate analytical code was used because the synthesis was conducted thematically and narratively

### 2.3 Prospects of SLR method in different fields

The SLR method is applied in several areas of knowledge including agricultural sciences, arts and humanities, behavioral and social sciences, business, economics and administrative sciences, chemical and materials engineering, commerce, computing and information technology, education, electrical, energy and environmental engineering, geo-sciences, health sciences, information and media studies, Islamic studies, law, life sciences, languages, industrial systems engineering, and pharmacy. It offers insightful implications to assist the bodies of knowledge and management. It provides evidence-based insights for informed policy making. The findings drawn from the SLR based studies are considered authentic, valid, effective, and reliable to refine existing systems and services (Iqbal et al., 2023; Khan et al., 2022; Tariq et al., 2026).

Investigators conducting investigations in several disciplines incorporate the methodology of SLR to collect and analyze studies available in worldwide published literature on specific topics. The SLR methodology assists to identify gaps in existing body of knowledge and practice on the basis of data-driven literature. Existing systems, policies, and services are reframed to empower and uplift different institutions (Ahmmad et al., 2025; Butt and Warraich, 2022; Jafree et al., 2021; Mahmood, 2016). In the SLR based studies, there are four stages that are called planning, selection, data extraction, and execution. These stages are elaborated as below systematically.

### 2.4 Planning

The research team executed planning to conduct an investigation on big data analytics and sentiment analysis for health misinformation detection: a systematic literature review of influencing factors, implementation challenges and a data-driven adoption framework. The following focused objectives of the study were developed:

- To identify the factors influencing big data analytics and sentiment analysis-based approaches for health misinformation detection.
- To reveal the challenges associated with the implementation of big data and sentiment-driven misinformation detection systems in healthcare information environments.

These objectives provided a roadmap to conduct the systematic review on the topic efficiently. Table 2 shows the inclusion and exclusion criteria that were considered for retrieving required studies (journal articles and conference proceedings).

**Table 2:** Inclusion and exclusion criteria.

**Sr. no**.	**Parameter**	Inclusion criteria	Exclusion criteria
1	Language	Studies published in English	Non-English documents
2	Coverage	Studies covering the study's objectives	Studies not addressing the study's objectives
3	Time span	Articles from January 1, 2015 to March 31, 2026	Articles prior to 2015
4	Variables	Studies on factors influencing big data analytics and sentiment analysis-based approaches for health misinformation detection	Studies without relevant variables
5	Content type	Peer-reviewed journal research papers and conference proceedings	Books, book reviews, dissertations, reports, gray literature, magazines, newspapers
6	Sources	Research indexed in key digital databases	Blogs, educational websites, institutional repositories, social media networks, search engines
7	Relevancy	Addressing at least one objective	Irrelevant to all objectives

### 2.5 Selection

The research team adopted various strategies to conduct a comprehensive search for the required documents. A comprehensive and structured search strategy was developed from the principal conceptual domains of the review: (i) big data analytics and sentiment analysis, and (ii) health misinformation detection, together with terms representing influencing factors, challenges, determinants, barriers, and drivers. Synonyms and closely related expressions were combined using the Boolean operators “AND” and “OR”. Database- and platform-specific syntax, field codes, controlled vocabulary, and search functions were adapted where necessary to accommodate the technical requirements of individual information sources. Eleven information sources were searched to maximize coverage across computing, health sciences, information science, and multidisciplinary research. These information sources included Google Scholar, Scopus, Web of Science, PubMed, MEDLINE, Ovid MEDLINE, ScienceDirect, Wiley InterScience, PLoS, Global Health, and MDPI. Google Scholar was treated as a scholarly search engine, whereas the remaining sources were searched through their respective bibliographic, publisher, and subject-specific platforms. All searches were conducted during June 2026.

The complete conceptual search strings used for each information source are presented in Table 3. Although the conceptual structure of the search was kept consistent across sources, field codes, controlled vocabulary, and platform-specific syntax were adapted where required. English-language studies published between 1 January 2015 and 31 March 2026 were considered according to the predefined eligibility criteria. The search process retrieved a total of 3,280 records. The use of multiple information sources was intended to improve the breadth of retrieval and reduce dependence on the indexing coverage of any single database or platform. Records identified through the different sources were subsequently subjected to duplication, title and abstract screening, and full-text eligibility assessment before the final set of included studies was established. To identify duplicate records, all retrieved references were imported into Mendeley Reference Manager, where duplicate entries were identified and checked before screening. The identified duplicate records were removed prior to title and abstract screening. This process resulted in the exclusion of 87 duplicate records from the initial 3,280 retrieved records.

**Table 3:** Complete search strings, information sources, search period, applied limits, and records retrieved.

Database	Search query applied	Limiters, keywords and controlled vocabulary	Results retrieved
Google Scholar	(“Big Data Analytics” OR “Big Data” OR “Data Analytics”) AND (“Sentiment Analysis” OR “Opinion Mining”) AND (“Health Misinformation” OR “Medical Misinformation” OR “Health Disinformation” OR “Fake Health News”) AND (“Factors” OR “Determinants” OR “Drivers” OR “Challenges” OR “Barriers”)	**Keywords:** big data analytics, sentiment analysis, health misinformation, influencing factors, implementation challenges **Language:** English **Years:** 2015–2026 **Document type:** peer-reviewed journal articles and conference proceedings	1,085
Scopus	TITLE-ABS-KEY((“Big Data Analytics” OR “Big Data”) AND (“Sentiment Analysis” OR “Opinion Mining”) AND (“Health Misinformation” OR “Medical Misinformation”) AND (“Factors” OR “Challenges”))	**Subject areas:** computer Science, Medicine, Health Sciences, Social Sciences **Language:** English **Years:** 2015–2026 **Document type:** peer-reviewed journal articles and conference proceedings	442
Web of Science	TS = ((“Big Data Analytics” OR “Big Data”) AND (“Sentiment Analysis” OR “Opinion Mining”) AND (“Health Misinformation” OR “Health Disinformation”) AND (“Factors” OR “Challenges”))	**Indexes:** SCI-E, SSCI, ESCI **Language:** English **Years:** 2015–2026 **Document type:** peer-reviewed journal articles and conference proceedings	318
PubMed	((“Big Data Analytics”[Title/Abstract] OR “Big Data”[Title/Abstract]) AND (“Sentiment Analysis”[Title/Abstract] OR “Opinion Mining”[Title/Abstract]) AND (“Health Misinformation”[Title/Abstract] OR “Medical Misinformation”[Title/Abstract]) AND (“Factors”[Title/Abstract] OR “Challenges”[Title/Abstract]))	**MeSH terms:** big data, artificial intelligence, health communication, social media **Language:** English **Years:** 2015–2026 **Document type:** peer-reviewed journal articles and conference proceedings	331
MEDLINE	(“Big Data Analytics” OR “Big Data”) AND (“Sentiment Analysis”) AND (“Health Misinformation”) AND (“Factors” OR “Challenges”)	**Controlled vocabulary:** MeSH **Language:** English **Years:** 2015–2026 **Document type:** peer-reviewed journal articles and conference proceedings	187
Ovid MEDLINE	exp Big Data/AND exp Sentiment Analysis/AND (Health Misinformation OR Medical Misinformation) AND (Factors OR Challenges)	**MeSH headings:** big data, health communication, information dissemination **Language:** English **Years:** 2015–2026 **Document type:** peer-reviewed journal articles and conference proceedings	142
ScienceDirect	(“Big Data Analytics” OR “Big Data”) AND (“Sentiment Analysis” OR “Opinion Mining”) AND (“Health Misinformation” OR “Health Disinformation”) AND (“Factors” OR “Challenges”)	**Subject areas:** medicine, computer science, health informatics, artificial intelligence **Language:** English **Years:** 2015–2026 **Document type:** peer-reviewed journal articles and conference proceedings	264
Wiley InterScience	(“Big Data Analytics”) AND (“Sentiment Analysis”) AND (“Health Misinformation” OR “Medical Misinformation”)	**Keywords:** big data analytics, healthcare information, artificial intelligence **Language:** English **Years:** 2015–2026 **Document type:** peer-reviewed journal articles and conference proceedings	82
PLoS	(“Big Data Analytics” OR “Big Data”) AND (“Sentiment Analysis”) AND (“Health Misinformation”)	**Keywords:** big data, sentiment analysis, public health, social media analytics **Language:** English **Years:** 2015–2026 **Document type:** peer-reviewed journal articles and conference proceedings	136
Global Health	(“Big Data Analytics”) AND (“Sentiment Analysis”) AND (“Health Misinformation”)	**Controlled vocabulary:** public health, digital health, health informatics **Language:** English **Years:** 2015–2026 **Document type:** peer-reviewed journal articles and conference proceedings	119
MDPI	(“Big Data Analytics” OR “Big Data”) AND (“Sentiment Analysis”) AND (“Health Misinformation”)	**Subject:** digital health, healthcare analytics, artificial intelligence **Language:** English **Years:** 2015–2026 **Document type:** peer-reviewed journal articles and conference proceedings	174

The applied strategies are displayed through Table 4.

**Table 4:** Search strategies for retrieving studies.

**Sr. no**.	**Strategy**	Objective
1	Derive themes from the study's objectives	Determine the key terms
2	Use title as core search phrase	Maintain strong alignment with the topic
3	Apply alternative spellings	Consider linguistic and regional differences
4	Selection of keywords according to research objectives	Ensure clarity and direction of the study
5	Develop a comprehensive research question	Provide a structured basis for systematic searching
6	Use keyword combinations from related studies	Maintain methodological consistency
7	Formulate equivalent terms and related expressions	Increase the scope of literature retrieval
8	Apply the Boolean operator “AND”	Restrict results to overlapping concepts
9	Employ the Boolean operator “OR”	Expand results to include alternative terms
10	Incorporate the Boolean operator “NOT”	Exclude irrelevant records

Table 3 show the search queries and filters that were employed in different digital to retrieve required studies.

Figure 1 shows the complete search process explicitly from the initial retrieval of the studies to the final selection of the most relevant core articles and conference proceedings. It shows that initially 3,280 documents were accessed. From 3,280 documents, 87 duplicate titles were excluded. Four hundred sixty-three studies were discarded due to ineligibility, 329 studies were skipped during the process of screening, and 558 documents were removed during full-text evaluation of the studies. Sixteen non-English language studies were also not included and 1,797 were excluded for not meeting the study's objectives. Finally, the 30 most relevant peer-reviewed research articles and conference proceedings were selected to conduct the systematic literature review (SLR) on the topic.

**Figure 1:**
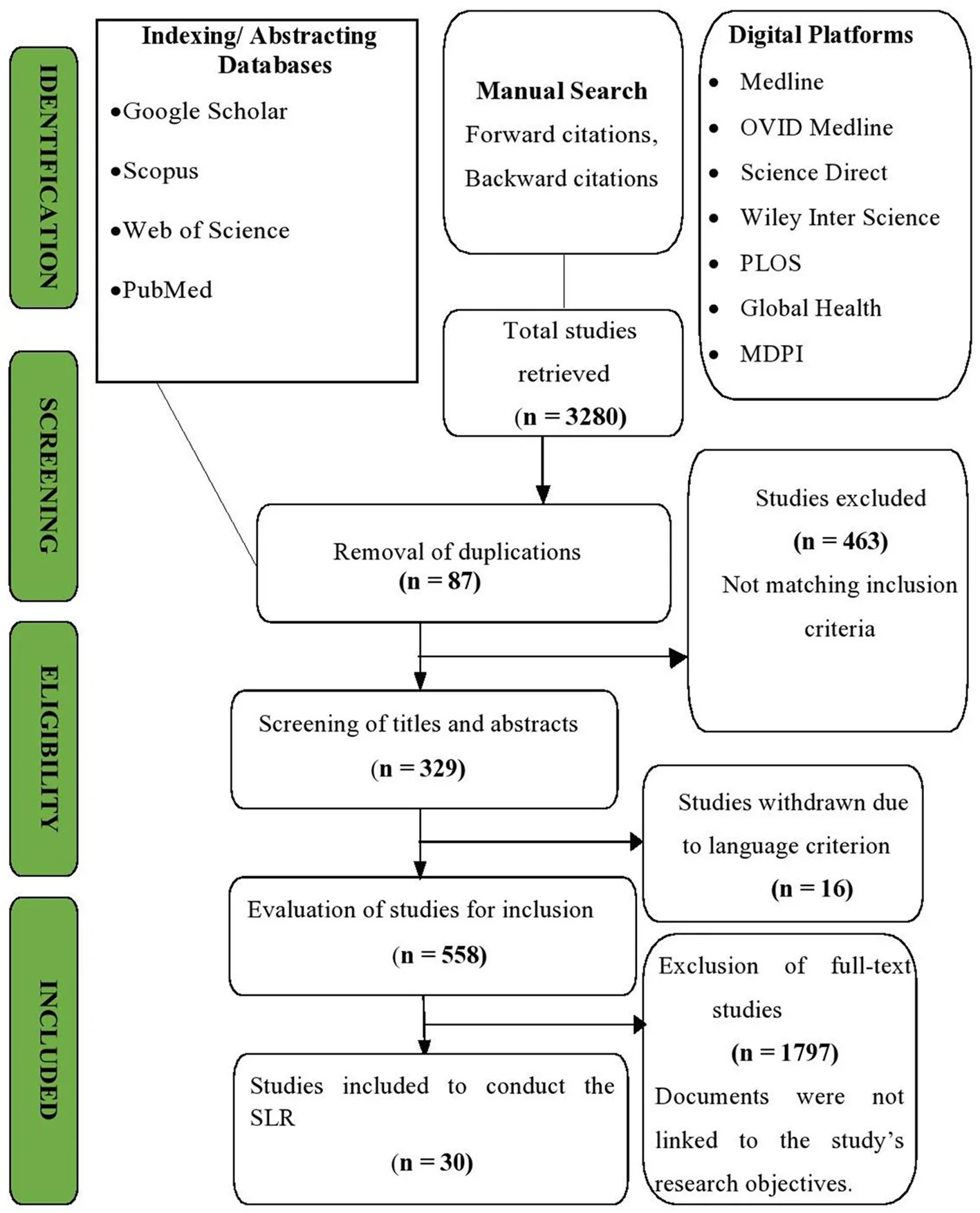
PRISMA four phase search diagram.

Although the search retrieved 3,280 records, only 30 studies satisfied all predefined eligibility criteria. The difference between the initial retrieval and final sample should not be interpreted as evidence that the review imposed an arbitrary sample-size restriction. Instead, the substantial reduction resulted from sequential duplication, title and abstract screening, full-text assessment, application of the language and publication-type criteria, and assessment of substantive relevance to the review objectives. The final sample therefore represents the body of evidence that met the pre-specified methodological and topical criteria rather than a predetermined number of studies. Nevertheless, the restrictive nature of the eligibility criteria may have excluded potentially relevant evidence. In particular, limiting the review to English-language peer-reviewed publications and conference proceedings may introduce language and publication bias, while differences in information-source coverage and indexing practices may contribute to selection bias. These limitations were considered when interpreting the findings and developing the data-driven adoption framework.

### 2.6 Reviewers, screening, study selection and quality assessment

The review process was conducted by the five authors of the study. Following the database searches conducted during June 2026, all retrieved records were compiled and organized for screening. The screening process was conducted in two sequential stages: (i) title and abstract screening, and (ii) full-text assessment against the predefined inclusion and exclusion criteria presented in Table 2. To minimize selection bias, the screening and eligibility decisions were independently undertaken by three reviewers from the author team. Any disagreements concerning the eligibility of a record were discussed among the reviewers and resolved through consensus. Where consensus could not be reached, the remaining two authors were consulted and a final decision was reached through discussion and consensus.

Following study selection, data were extracted using the structured extraction framework presented in Table 5. The extracted information included bibliographic characteristics, geographical context, big data analytics and sentiment-analysis approaches, influencing factors, implementation challenges, and other information relevant to the review objectives. Data extraction was independently undertaken by four reviewers, with discrepancies checked, discussed, and resolved through consensus. The methodological quality of the 30 included studies was assessed using the 10-item CASP-informed critical appraisal framework presented in Table 6. Quality appraisal was conducted by three reviewers. Any differences in appraisal judgments were discussed and resolved through consensus. The quality assessment was used to contextualize the synthesized findings. Studies with methodological limitations were not automatically excluded when they met the predefined eligibility criteria; instead, their methodological strengths and limitations were considered when interpreting the strength and consistency of the evidence.

**Table 5:** Datasets extracted through 30 studies.

Reference	Location	Journal/ conference	Factors influencing BDA and SA	Challenges associated with BDA and SA adoption
Ahmed and Ahmed (2023)	India	Microprocessors and microsystems	•Multi-class machine learning classification framework •Communication platform-specific data analytics •Adaptive sentiment classification models •Intelligent feature representation for text classification •Cross-platform analytics for misinformation detection	•Variability in data characteristics across communication platforms •Difficulty in processing multilingual and informal textual content •Maintaining consistent model performance in dynamic online environments
Alarabid et al. (2026)	UAE	International conference on information system design and intelligent applications	•Large language model (LLM)-based contextual reasoning •Prompt engineering for misinformation classification •Transfer learning from pre-trained foundation models •Few-shot and zero-shot learning capabilities •Fine-tuning on domain-specific public health datasets	•Hallucination and unreliable LLM-generated outputs •High computational and infrastructure costs for LLM deployment •Limited explainability of LLM-based misinformation decisions
Alonso et al. (2021)	Spain	Electronics	•Emotion-aware sentiment feature extraction •Polarity intensity analysis of textual content •Integration of sentiment features with linguistic features •Transformer-based contextual text representations •Multi-feature fusion for fake news classification	•Weak discriminative power of sentiment features when used alone •Difficulty in detecting implicit emotional expressions •Variability of sentiment across different fake news domains
Arias et al. (2022)	Panama	IEEE access	•Multi-platform social media data acquisition •Health-specific sentiment lexicon development •Named entity recognition for health information extraction •Aspect-based sentiment analysis for health topics •Public opinion mining for health decision support	•Scarcity of domain-specific annotated health sentiment datasets •Difficulty in detecting implicit opinions and emotions •Limited generalizability of sentiment models across health domains
Arora et al. (2026)	India	Discover mental health	•Transformer-based semantic understanding of health content •Mental health-aware sentiment classification •Attention mechanisms for contextual feature extraction •Cross-domain learning for misinformation detection •Explainable AI techniques for model interpretation	•Distinguishing misinformation from genuine mental health expressions •Limited availability of labeled mental health misinformation datasets •Ethical concerns regarding sensitive mental health data analysis
Azzaoui et al. (2021)	Republic of Korea	Sustainable cities and society	•Social network service (SNS) data integration •Real-time sentiment trend monitoring •Geospatial analytics for outbreak surveillance •IoT-enabled big data ecosystem integration •Predictive analytics for early public health response	•Integration of heterogeneous IoT and social media data •Ensuring real-time processing of massive streaming datasets •Data privacy and security concerns in smart city environments
Babar et al. (2024)	Saudi Arabia	IEEE transactions on computational social systems	•Deep neural network-based feature learning •Real-time streaming data analytics •Distributed computing for large-scale data processing •Automated feature selection and dimensionality optimization •Continuous model updating with incoming data	•Latency constraints in real-time misinformation detection •High computational complexity of deep neural networks •Maintaining model robustness against evolving fake news content
Balshetwar et al. (2023)	India	Multimedia tools and applications	•Comparative evaluation of multiple classifier algorithms •Sentiment score-based feature engineering •Feature selection for classifier optimization •Text vectorization using TF–IDF representations •Social media text mining for misinformation detection	•Misclassification of mixed and neutral sentiments •Sensitivity of classifier performance to feature selection •Reduced detection accuracy for short and noisy social media posts
Cavus et al. (2025)	Cyprus	Applied sciences	•Cloud-based scalable analytics infrastructure •BERT-based contextual sentiment representation •Automated tweet preprocessing pipeline •GPU-enabled model training and inference •End-to-end cloud deployment for sentiment analytics	•High computational demands of transformer-based models •Dependence on cloud resources for large-scale processing •Reduced performance on rapidly evolving social media language
Chatta et al. (2026)	Algeria	International journal of informatics and applied mathematics	•Arabic-specific text preprocessing and normalization •Sentiment-enhanced feature representation for fake news detection •Deep learning models for Arabic text classification •Word embedding techniques for Arabic semantic analysis •Language-specific feature fusion for misinformation detection	•Complex morphology and dialectal variation in Arabic text •Scarcity of annotated Arabic misinformation datasets •Difficulty in handling code-switching and informal Arabic expressions
Cynthia et al. (2026)	Cameroon	Health informatics journal	•Hugging Face transformer models for sentiment classification •Cross-platform social media data integration (X and Facebook) •Vaccine perception mining from user-generated content •Domain-adapted transformer models for public health analytics •Comparative sentiment analysis across social media platforms	•Platform-specific differences in user engagement and sentiment •Presence of multilingual and code-mixed social media content •Sampling bias due to unequal representation of social media users
Di Sotto and Viviani (2022)	Italy	International journal of environmental research and public health	•Availability of large-scale social media data •High-quality data preprocessing and cleaning •Advanced natural language processing (NLP) techniques •Machine learning-based feature extraction and classification •Integration of multimodal data sources for misinformation detection	•High volume and heterogeneity of social media data •Rapid evolution of health misinformation narratives •Limited availability of high-quality labeled datasets
Feng et al. (2025)	China	INQUIRY: the journal of health care organization, provision, and financing	•Knowledge graph integration for contextual reasoning •Multi-source health information fusion •Transformer-based language models for text understanding •Domain-specific health knowledge incorporation •Human–AI collaborative verification mechanisms	•Limited availability of annotated health misinformation datasets •Poor generalizability across different health domains and platforms •Detection of multimodal and AI-generated health misinformation
George and Baskar (2024)	India	Partners universal international innovation journal	•Scalable data mining frameworks for social media analytics •Integration of structured and unstructured data •Predictive analytics for trend identification •Interactive visual analytics for decision support •Automated social media data harvesting	•Ensuring data veracity in large-scale social media analytics •Managing concept drift in continuously evolving data •Compliance with ethical and regulatory requirements for social media data use
Hussain et al. (2023)	Canada	International conference on wireless and mobile computing, networking and communications	•AI-driven decision support for public health authorities •Automated misinformation risk scoring •Real-time public health surveillance dashboards •Integration of analytical outputs for policy decision-making •Continuous monitoring of misinformation outbreaks	•Translating analytical results into timely public health actions •Scalability of surveillance systems during health emergencies •Integration of AI-based monitoring within government health infrastructures
Hussain et al. (2023)	Canada	European journal of public health	•Automated data extraction through Twitter V2 API •Pre-trained AI models for misinformation detection •Dashboard-based analytics and data visualization •Anomaly and risk detection for misinformation trends •Multi-platform scalability for future surveillance	•Lack of comprehensive infodemic surveillance systems •Limited support for non-Western language content •Continuous monitoring of rapidly growing social media misinformation streams
Liu et al. (2019)	China	IEEE access	•User credibility and profile characteristics •Temporal propagation pattern analysis •Social interaction features (reposts, comments, likes) •Linguistic and semantic feature extraction •Ensemble learning for misinformation classification	•Language-specific processing complexities in Chinese social media •Early-stage detection of rapidly spreading misinformation •Limited transferability of detection models across social media platforms
Malek et al. (2026)	USA	Journal of medical internet research	•Automated thematic analysis using large language models •Retrieval-augmented generation (RAG) for evidence-grounded analysis •Health misinformation inoculation through AI-generated messaging •Context-aware claim verification using LLMs •Human-in-the-loop validation for model refinement	•Risk of hallucinated or unsupported health recommendations •Dependence on high-quality external knowledge sources for reliable outputs •Ensuring factual consistency and trustworthiness of AI-generated interventions
Cano-Marin et al. (2023)	Spain	Technological forecasting and social change	•Social network analysis (SNA) for information diffusion •Graph analytics for misinformation pattern discovery •Natural language processing of user-generated content •Machine learning-based predictive analytics •Large-scale Twitter data availability for health surveillance	•Presence of fake news and rumors affecting data reliability •Verification of information credibility in social media datasets •Bias and representativeness issues in Twitter-derived data
Morita et al. (2023)	Canada	Journal of medical internet research	•Real-time social media data acquisition via APIs •Expert-validated training datasets •Topic modeling using Latent Dirichlet Allocation (LDA) •Cloud-based big data storage and visualization (elastic Stack) •Scalable end-to-end big data pipeline architecture	•Dependence on expert-labeled data for model training •Integration of multiple analytics components within a unified ecosystem •Achieving accurate real-time misinformation classification at scale
Kabir (2022)	USA	Online media and global communication	•Balanced Random Forest for imbalanced sentiment datasets •Comparative evaluation of multiple machine learning classifiers •YouTube user comments as a large-scale data source •LDA-based topic modeling for thematic extraction •Confusion matrix and accuracy-based model evaluation	•Class imbalance in social media sentiment data •Noisy and unstructured user-generated comments •Persistent negative attitudes despite misinformation correction efforts
Kansara and Adhvaryu (2022)	India	International conference on automation, computing and renewable systems	•Near real-time analytics for misinformation detection •Batch and stream data processing integration •Distributed storage for high-volume social media data •Sentiment-driven event monitoring •Data ingestion from multiple online platforms	•Synchronizing real-time data collection and processing •Managing data inconsistency across heterogeneous sources •Balancing detection speed with analytical accuracy
Kansara and Adhvaryu (2024)	India	SN computer science	•Distributed big data processing using Apache Spark and Hadoop •Lexicon-based and hybrid sentiment analysis approaches •Metadata feature extraction for fake news detection •Dimensionality reduction for high-dimensional datasets •Cross-validation for robust model performance assessment	•High dimensionality and complexity of big data •Detection of sarcastic and misleading textual content •Lack of standardized benchmark datasets for model comparison
Marques et al. (2022)	Brazil	International journal of environmental research and public health	•Large-scale online news content collection •Automated sentiment polarity classification •Temporal monitoring of public sentiment trends •Integration of news analytics for public health surveillance •Data visualization for sentiment pattern interpretation	•Variation in sentiment across different news sources •Difficulty in interpreting context-dependent health narratives •Influence of media framing on sentiment analysis accuracy
Shahzad et al. (2022)	Pakistan	Sustainability	•Context-aware feature extraction for misinformation detection •User behavior and engagement analytics •Source credibility assessment mechanisms •Semantic context modeling using NLP •Multi-dimensional feature integration for classification	•Limited contextual understanding of misleading content •Dynamic evolution of fake news dissemination patterns •Absence of unified context-aware detection frameworks
Sharma et al. (2025)	India	International conference on electrical engineering and informatics	•High-quality and reliable datasets •Robust data integration from heterogeneous sources •Advanced data preprocessing and feature engineering •Scalable big data analytics infrastructure •Continuous model training and performance optimization	•Poor data quality and noisy datasets •Scalability issues with rapidly growing data volumes •Maintaining detection accuracy across diverse misinformation patterns
Taha (2025)	UAE	Journal of big data	•Distributed stream processing for real-time analytics •Cloud and edge computing integration •Advanced NLP pipelines for large-scale text analysis •Interoperability among heterogeneous data sources •Security-aware big data architecture design	•Data privacy and cybersecurity vulnerabilities •Managing heterogeneous and high-velocity data streams •Resource-intensive processing and storage requirements
Villanueva-Miranda et al. (2025)	USA	Frontiers in public health	•Adoption of deep learning and large language models (LLMs) •Domain-specific model adaptation for health contexts •Rigorous model validation and performance evaluation •Explainable and interpretable AI techniques •Multilingual sentiment analysis capabilities	•High computational and resource requirements •Limited model interpretability and transparency •Ethical concerns related to privacy, bias, and responsible AI deployment
Wreyford et al. (2026)	Australia	Nursing reports	•Patient-generated health data integration •Patient voice mining from digital health platforms •Longitudinal sentiment tracking of treatment experiences •Patient-centered NLP for clinical narratives •Sentiment-informed prediction of treatment adherence	•Heterogeneity of patient-reported health narratives •Privacy and confidentiality of patient-generated data •Limited clinical validation of sentiment-based predictive models
Zamir et al. (2024)	Mexico	PLoS ONE	•Hybrid machine learning and deep learning models •Word embedding techniques for semantic representation •Effective text normalization and tokenization •Hyperparameter tuning for model optimization •Comprehensive performance evaluation using multiple metrics	•Detection of context-dependent and ambiguous sentiments •High computational cost of deep learning models •Performance degradation due to evolving misinformation patterns

**Table 6:** CASP checklist for quality appraisal.

QA ID	Checklist questions
QA 1	Are study's objectives clear?
QA 2	Has an appropriate research methodology been applied?
QA 3	How sampling methods have been recruited?
QA 4	Has required data been collected through rigorous techniques?
QA 5	Have confounding and bias factors been considered?
QA 6	Has the gathered data been analyzed efficiently?
QA 7	Are results clear and credible?
QA 8	Is the study clear overall?
QA 9	Is the study rigorous throughout?
QA 10	Are all the study's sections relevant to one another?

Yes = 2, partially = 1, no = 0, barely = 0.5, satisfactorily = 1.5.

**Source:** Shahzad and Tariq (2025).

### 2.7 Justification of the final sample and potential selection bias

Although the search retrieved 3,280 records, however the final sample consisted of only 30 studies that satisfied all predefined eligibility criteria. The relatively small final sample was not determined by an arbitrary sample-size target. It resulted from the systematic application of the pre-specified inclusion and exclusion criteria to the complete set of retrieved records. The broad initial retrieval indicates the use of multiple information sources and deliberately inclusive search terms, whereas the substantially smaller final sample shows the specific focus of the review on big data analytics and sentiment analysis in relation to health misinformation detection, including relevant influencing factors and implementation challenges. The studies were not retained merely because they were conceptually relevant to the topic. Records addressing general health misinformation, general social-media analytics, unrelated applications of big data or sentiment analysis, and topics outside the predefined review objectives were excluded when they failed to satisfy the eligibility criteria.

The selection process may be affected by potential publication and selection bias. The review was restricted to English-language, peer-reviewed journal articles and conference proceedings and therefore may have excluded relevant evidence published in other languages or in gray literature, reports, dissertations, books, and other non-indexed sources. In addition, differences in database coverage and indexing practices may have influenced which studies were retrievable. These limitations were considered while interpreting the synthesized evidence. Consequently, the findings should be considered as representing the eligible indexed literature retrieved through the specified search strategy rather than the entire universe of research on big data analytics, sentiment analysis, and health misinformation detection.

### 2.8 Data extraction and quality appraisal

A structured scorekeeping method was applied to evaluate the methodological quality, relevance, and reporting rigor of the included studies. A predefined 10-item CASP-informed quality assessment framework was developed and applied consistently to all 30 included studies. The assessment criteria examined the clarity of study objectives, appropriateness of the research methodology, sampling procedures, rigor of data collection, consideration of confounding and bias, appropriateness of data analysis, clarity and credibility of results, study clarity, and consistency among the different sections of the study.

Each study was assessed against all predefined criteria. Responses were recorded using a five-category scale: “Yes”, “Satisfactorily”, “Partially”, “Barely”, and “No”. To facilitate systematic comparison and synthesis, the qualitative judgments were converted into numerical scores. A response of “Yes” received 2 points, “Satisfactorily” received 1.5 points, “Partially” received 1 point, “Barely” received 0.5 points, and “No” received 0 points. Thus, each study could obtain a maximum score of 20 points across the 10 assessment criteria. The overall quality percentage was calculated by dividing the total score obtained by the maximum possible score and multiplying the result by 100.

The individual quality-assessment results for all 30 included studies are presented in Table 7. The scoring procedure enabled the methodological strengths and limitations of the included studies to be compared systematically and provided a transparent basis for interpreting the evidence synthesized in the review. As the included literature comprised different types of studies, including empirical, methodological, review, and technical studies, so, the appraisal was applied as a CASP-informed framework rather than being described as a verbatim application of a single design-specific CASP checklist.

**Table 7:** Quality assessment of the 30 included studies.

**No**.	**Reference**	QA1	QA2	QA3	QA4	QA5	QA6	QA7	QA8	QA9	QA10	Total/20	Quality %
1	Ahmed and Ahmed (2023)	2	2	1.5	2	1	2	2	2	1.5	2	18.0	90%
2	Alarabid et al. (2026)	2	2	1	1.5	1	2	2	2	1.5	2	17.0	85%
3	Alonso et al. (2021)	2	1.5	1	1.5	1	2	2	2	1.5	2	16.5	82.5%
4	Arias et al. (2022)	2	2	1.5	2	1	2	2	2	1.5	2	18.0	90%
5	Arora et al. (2026)	2	2	1.5	2	1.5	2	2	2	2	2	19.0	95%
6	Azzaoui et al. (2021)	2	2	1.5	2	1	2	2	2	1.5	2	18.0	90%
7	Babar et al. (2023)	2	2	1.5	2	1.5	2	2	2	2	2	19.0	95%
8	Balshetwar et al. (2023)	2	1.5	1.5	1.5	0.5	1.5	2	1.5	1.5	1.5	15.0	75%
9	Cavus et al. (2025)	2	2	1.5	2	1	2	2	2	1.5	2	18.0	90%
10	Chatta et al. (2026)	2	2	1.5	2	1	2	2	2	1.5	2	18.0	90%
11	Cynthia et al. (2026)	2	2	1.5	2	1	2	2	2	1.5	2	18.0	90%
12	Di Sotto and Viviani (2022)	2	1.5	0.5	1	1	1.5	2	2	1.5	1.5	14.5	72.5%
13	Feng et al. (2025)	2	1.5	0.5	1	1	1.5	2	2	1.5	2	15.0	75%
14	George and Baskar (2024)	2	1.5	0.5	1	1	1.5	2	2	1.5	1.5	14.5	72.5%
15	Hussain et al. (2023)	2	2	1.5	2	1	2	2	2	1.5	2	18.0	90%
16	Hussain et al. (2023)	2	2	1.5	2	1	2	2	2	1.5	2	18.0	90%
17	Liu et al. (2019)	2	2	2	2	1	2	2	2	2	2	19.0	95%
18	Malek et al. (2026)	2	2	1.5	2	1.5	2	2	2	2	2	19.0	95%
19	Cano-Marin et al. (2023)	2	1.5	0.5	1	1	1.5	2	2	1.5	2	15.0	75%
20	Morita et al. (2023)	2	2	1.5	2	1.5	2	2	2	2	2	19.0	95%
21	Kabir (2022)	2	2	1.5	2	1	2	2	2	1.5	2	18.0	90%
22	Kansara and Adhvaryu (2022)	2	2	1.5	2	1	2	2	2	1.5	2	18.0	90%
23	Kansara and Adhvaryu (2024)	2	1.5	0.5	1.5	1	1.5	2	2	1.5	2	15.5	77.5%
24	Marques et al. (2022)	2	2	1.5	2	1	2	2	2	1.5	2	18.0	90%
25	Shahzad et al. (2022)	2	1.5	0.5	1	1	1.5	2	2	1.5	1.5	14.5	72.5%
26	Sharma et al. (2025)	2	2	1.5	2	1.5	2	2	2	1.5	2	18.5	92.5%
27	Taha (2025)	2	1.5	0.5	1	1	1.5	2	2	1.5	1.5	14.5	72.5%
28	Villanueva-Miranda et al. (2025)	2	1.5	0.5	1	1	1.5	2	2	1.5	2	15.0	75%
29	Wreyford et al. (2026)	2	1.5	0.5	1	1	1.5	2	2	1.5	2	15.0	75%
30	Zamir et al. (2024)	2	2	1.5	2	1.5	2	2	2	2	2	19.0	95%

Scoring key: QA1–QA10: yes = 2; satisfactorily = 1.5; partially = 1; barely = 0.5; no = 0. Maximum = 20 points.

The appraisal criteria were interpreted in relation to the methodological characteristics and purpose of each study rather than applying identical assumptions mechanically across all study designs. For empirical and observational studies, criteria concerning research objectives, sampling or participant selection, data collection, potential bias or confounding, data analysis, and credibility of findings were assessed according to the procedures reported by the authors. For technical, computational, and algorithmic studies, the corresponding criteria were interpreted in relation to the suitability of data sources, analytical techniques, model development, validation procedures, performance assessment, and reporting transparency. For review, scoping, and scientometric studies, the criteria were interpreted in relation to the clarity of review objectives, transparency of evidence identification and selection, appropriateness of the review or analytical methodology, synthesis procedures, and reporting rigor. For conference-based studies, the same domains were applied according to the methodological information available in the published proceedings paper. This design-sensitive approach retained the same 10 appraisal domains across the evidence base to support comparability while allowing their interpretation to reflect the methodological characteristics of individual studies.

All included studies were independently evaluated by at least two reviewers from the author team to enhance rigor and consistency in the quality-appraisal process. Any discrepancies in scoring were recorded and subsequently resolved through discussion and consensus. Where disagreement persisted, a third reviewer was consulted to make the final decision and minimize the possibility of individual reviewer bias. Inter-rater agreement was assessed using Cohen's Kappa coefficient. A value of κ > 0.75 was interpreted as indicating a high level of agreement among the reviewers in applying the predefined appraisal criteria. This procedure was intended to enhance the transparency, reproducibility, and methodological rigor of the quality-assessment process.

For interpretation purposes, predefined thresholds were established for the methodological quality scores. Studies scoring 75%−100% were classified as having high methodological quality. Studies scoring 50%−74.9% were classified as having moderate methodological quality, and studies scoring below 50% were classified as having low methodological quality. These thresholds were defined operationally for the present review to facilitate consistent interpretation of the appraisal results. The quality categories were used to contextualize and interpret the synthesized evidence rather than as automatic exclusion criteria. Thus, studies with methodological limitations were retained when they satisfied the predefined eligibility criteria, while their methodological quality was considered when interpreting the consistency and strength of the reported evidence.

The quality assessment was used primarily to contextualize the synthesized evidence. Studies that satisfied the predefined eligibility criteria were not automatically excluded due to methodological limitations. The identified strengths and limitations were considered while interpreting the consistency and robustness of the evidence and while developing the data-driven adoption framework. Table 7 displays quality mapping scores of the 30 included studies.

For mapping the selected research articles and conference proceedings systematically (*n* = 30), a structured form (Table 5) was formulated. The form included the following components:

- Authors with publishing years.
- Geographical territories.
- Journals.
- Factors influencing big data analytics and sentiment analysis-based approaches for health misinformation detection.
- Challenges associated with the implementation of big data and sentiment-driven misinformation detection systems in healthcare information environments.

### 2.9 Execution and thematic synthesis

The extracted evidence was synthesized using a structured thematic synthesis approach aligned with the two research objectives. The synthesis was conducted in four sequential stages. Firstly, the findings extracted from the 30 included studies were reviewed to identify recurring concepts relating to big data analytics (BDA), sentiment analysis (SA), health misinformation detection, influencing factors, and implementation challenges. Secondly, relevant findings were coded according to their substantive meaning rather than according to the terminology used by individual studies. Thirdly, conceptually related codes were compared, grouped, and organized into descriptive themes representing recurring technological, data-related, organizational, contextual, and implementation-related factors and challenges. Fourthly, the descriptive themes were compared across studies and developed into higher-level analytical categories that directly addressed the research objectives and informed the proposed data-driven adoption framework.

During synthesis, similarities and differences across studies were considered related to research design, geographical context, data sources, analytical approaches, application domains, and methodological quality. Where different studies used different terminology for conceptually similar factors, related concepts were consolidated into common analytical themes while preserving their substantive meaning. Conversely, conceptually distinct factors were retained as separate themes even when they occurred within the same studies. The methodological quality scores were also considered while interpreting the consistency and robustness of the supporting evidence. Because the included studies differed substantially in research design, measures, datasets, contexts, and outcomes, statistical pooling was not considered appropriate. The findings were therefore synthesized narratively and thematically rather than through meta-analysis.

### 2.10 Evidence traceability and support for major factors

To strengthen evidence traceability, each major factor and implementation challenge identified through the thematic synthesis was linked to the individual included studies from which supporting evidence was extracted. The Results section presents the major themes with citations to the contributing studies. The number and methodological characteristics of supporting studies were considered during interpretation; however, study frequency alone was not treated as an indicator of evidential strength. In the last phase, the extracted datasets were analyzed thematically for addressing the study's research objectives. The following components were considered for interpretation:

- Geographical distribution of studies.
- Research methods applied in the selected studies.
- Factors influencing the adoption of big data analytics (BDA) and sentiment analysis (SA) for health misinformation detection.
- Challenges to incorporate BDA and SA.
- Discussion.
- Framework to adopt BDA and SA for health misinformation detection on social media.
- Implications.
- Limitations and Future Research Directions.
- Conclusion.

## 3 Results

Table 5 displays an overview of the selected studies. It shows information about authors with publishing years geographical territories, and journals. It also manifests information about factors influencing big data analytics and sentiment analysis-based approaches for health misinformation detection and challenges associated with the implementation of big data and sentiment-driven misinformation detection systems in healthcare information environments.

### 3.1 Geographical distribution of studies

Findings of the study showed that most of the included studies were produced from India (*n* = 7). Both USA and Canada contributed three studies each related to factors influencing big data analytics and sentiment analysis based health misinformation detection. The results displayed that the included studies (*n* = 30) were published from 17 different regions of the world. Figure 2 displays geographical distribution of the selected studies.

**Figure 2:**
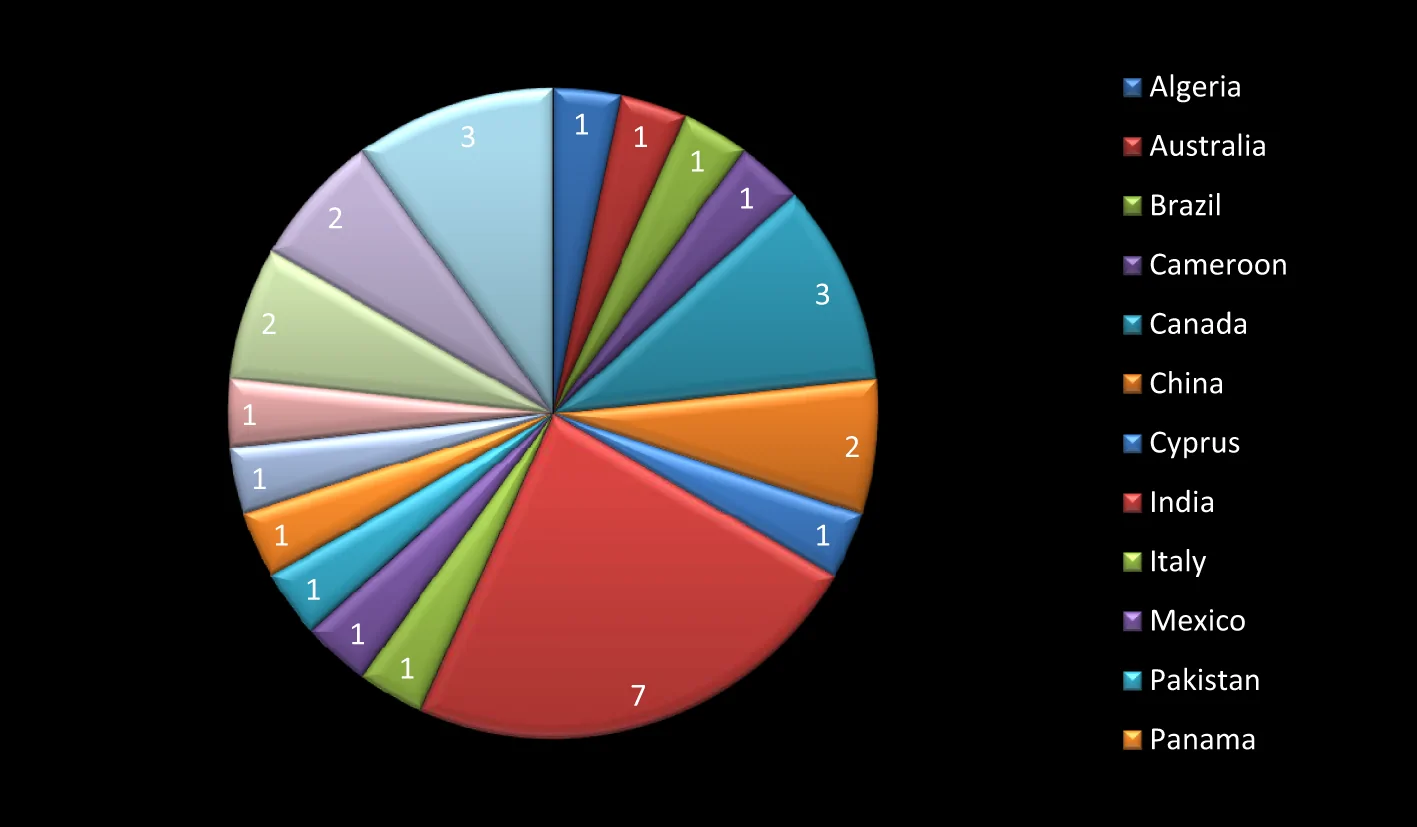
Geographical distribution of the selected studies.

### 3.2 Research methods applied in the selected studies

The findings showed that the experimental method was the most applied methodology in the included studies (*n* = 10). Literature review methodology was employed in five studies whereas content analysis method was applied in four studies. In the included studies (*n* = 30), 10 different research methodologies were used. Figure 3 shows the research methods applied in the selected studies.

**Figure 3:**
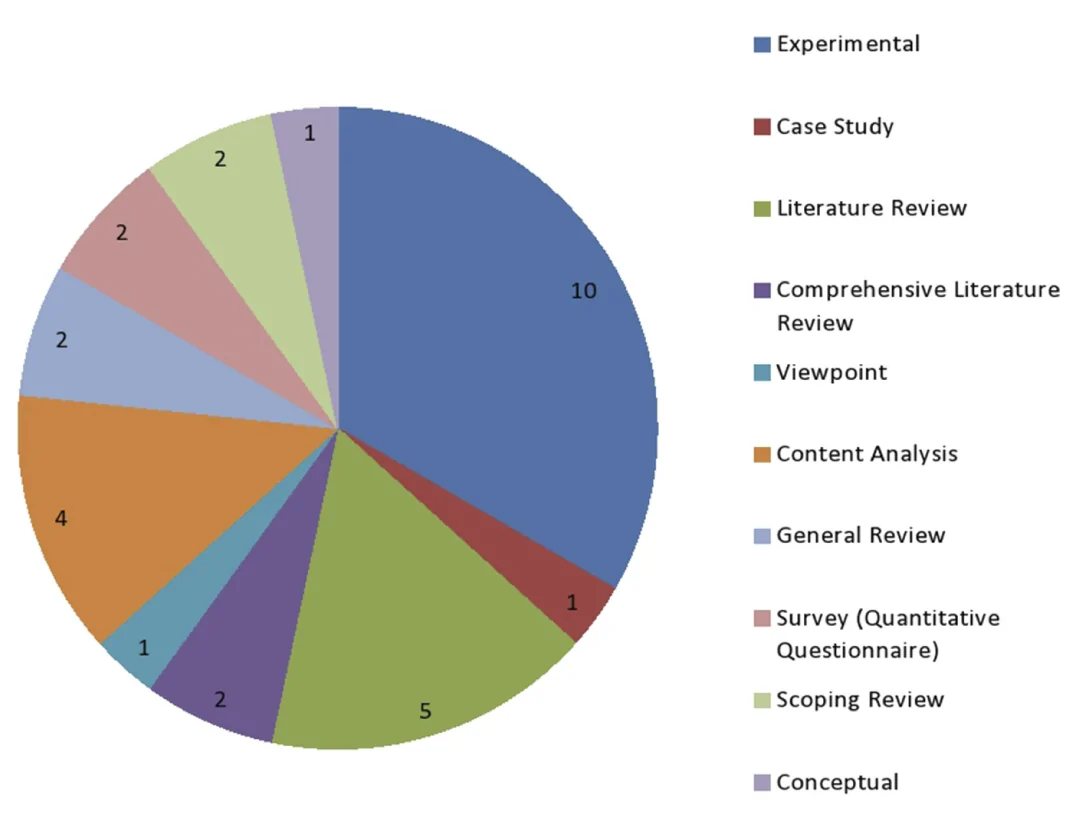
Research methods applied in the selected studies.

### 3.3 Factors influencing big data analytics and sentiment analysis-based health misinformation detection

The findings indicate that advanced deep learning architectures, feature engineering, sentiment-derived signals, real-time analytics, and human-centered verification are important components influencing data-driven health misinformation detection. Importantly, sentiment analysis was identified as an auxiliary analytical signal that can capture emotional, attitudinal, and opinion-related characteristics of health-related content. It does not independently establish whether information is factual or misleading. Reliable misinformation detection therefore requires the integration of sentiment signals with contextual semantics, source and evidence assessment, behavioral or temporal patterns, and other verification mechanisms. These factors are interpreted as follows alternately:

#### 3.3.1 Advanced deep learning architectures

The results showed that advanced deep learning architecture is a key factor to identify health misinformation through deep neural representation learning techniques (Alarabid et al., 2026; Arora et al., 2026; Cavus et al., 2025; Villanueva-Miranda et al., 2025). Large language models contributed contextual reasoning through domain specific fine tuning to reduce reliance on manual datasets (Alarabid et al., 2026; Malek et al., 2026). Machine learning frameworks enhanced semantic abstraction that proved fruitful in strengthening the identification of misinformation in healthcare domain (Babar et al., 2024; Zamir et al., 2024). Semantic representations played a vital role in solving conventional lexical complications (Alonso et al., 2021; Arora et al., 2026; Cavus et al., 2025). Attention mechanisms proved impactful in enhancing contextual feature extraction through priority on clinical relevant textual signals and reduced risks of fake content generated by users (Arora et al., 2026). Multi classification strategies attained rigor by controlling heterogeneous misinformation patterns in healthcare industry (Ahmed and Ahmed, 2023; Kabir, 2022; Zamir et al., 2024). Advanced deep learning architectures significantly detects health misinformation across diverse health environments by improving adaptive learning, contextual reasoning, and semantic generalization (Alarabid et al., 2026; Arora et al., 2026; Villanueva-Miranda et al., 2025).

#### 3.3.2 Feature engineering

The review demonstrated that feature engineering is a valuable factor that positively assists to identify health misinformation. Authentic data, integration, and engineering of underlying datasets proved valuable in detecting health misinformation (Di Sotto and Viviani, 2022; Sharma et al., 2025). High quality data improved classification performance across transformer based frameworks and machine learning models (Babar et al., 2023; Cavus et al., 2025; Di Sotto and Viviani, 2022; Zamir et al., 2024). Feature engineering enhanced analytical coverage through heterogeneous users' interactions at social media and digital health environments (Arias et al., 2022; Cynthia et al., 2026; Morita et al., 2023). Different feature-engineering approaches, including sentiment-score extraction, optimization, and automatic feature selection, can reduce redundant information and provide complementary affective signals for broader misinformation classification (Babar et al., 2024; Balshetwar et al., 2023). The integration of structured and unstructured datasets enabled predictive analytics for detecting health misinformation (George and Baskar, 2024; Sharma et al., 2025). Entity recognition, domain specific health lexicons and semantic normalization accurately extracted health relevant misinformation (Arias et al., 2022; Shahzad et al., 2022). Feature engineering serves as a foundational enabler to efficiently operationalize health misinformation identification (Di Sotto and Viviani, 2022; George and Baskar, 2024; Sharma et al., 2025).

#### 3.3.3 Sentiment-derived intelligence as an auxiliary signal

The review identified sentiment-derived information as an important auxiliary signal within broader health misinformation analytics. Sentiment analysis covers emotional polarity, attitudes, opinions, and affective characteristics expressed in health-related digital content. These signals can complement semantic and contextual representations when analyzing potentially misleading information; however, sentiment polarity alone does not establish the factual accuracy of a health claim. Sentiment analysis integrated with contextual semantic modeling can contribute to misinformation classification by providing information about emotional and attitudinal patterns that may be relevant to the communication and dissemination of health-related content (Alonso et al., 2021; Shahzad et al., 2022). Emotion-aware features may assist in characterizing potentially manipulative or highly affective communication, but such characteristics require interpretation alongside other evidence rather than being treated as direct indicators of misinformation (Alonso et al., 2021).

Sentiment-derived information can also enhance topic-sensitive analysis by helping characterize public reactions to health-related information and identifying patterns of emotional response across digital platforms (Arias et al., 2022). Similarly, health-aware sentiment classification can help distinguish different forms of emotional expression, including potentially harmful or misleading narratives and legitimate expressions of psychological stress; nevertheless, such distinctions require contextual interpretation rather than sentiment polarity alone (Arora et al., 2026). Linguistic and semantic representations remain necessary because misinformation may be communicated through neutral, positive, or negative sentiment. Consequently, contextual language modeling, domain-specific knowledge, user behavior, source characteristics, and other evidence can provide information that sentiment analysis cannot independently establish (Liu et al., 2019; Shahzad et al., 2022). Multilingual and culturally diverse expressions further demonstrate the need to interpret sentiment within its linguistic and social context (Chatta et al., 2026; Zamir et al., 2024). On the whole, the evidence suggests that sentiment analysis is analytically valuable when incorporated with contextual semantics, user behavior, domain-specific health knowledge, and verification mechanisms. It should therefore be considered as a complementary analytical signal within a multidimensional misinformation detection system rather than as a standalone mechanism for determining whether health information is true or false.

#### 3.3.4 Real-time analytics

The findings showed that real time analytics are an important factor that determines operational effectiveness in health misinformation domain (Kansara and Adhvaryu, 2024; Taha, 2025). Cloud based analytics efficiently processed large volumes of health datasets to identify patterns of health misinformation on social media streams (Cavus et al., 2025; Kansara and Adhvaryu, 2024; Taha, 2025). Real time streaming analytics supported immediate identification of health misinformation to effectively respond to public health events (Babar et al., 2024; Azzaoui et al., 2021). Real time integration of steam processing increased analytical capability while maintaining monitoring of fake health information across various digital platforms (Kansara and Adhvaryu, 2022). Cloud based storage and visualization assisted in training infrastructures for large scale sentiment analysis (Cavus et al., 2025; Morita et al., 2023). Internet of Things (IoT) powered ecosystems expanded the scope of social sentiment patterns to ensure public health safety (Azzaoui et al., 2021). The results show that scalable infrastructure is a strategic enabler that facilitates in real time health misinformation detection (Kansara and Adhvaryu, 2024; Morita et al., 2023; Taha, 2025).

#### 3.3.5 Human-centered verification

The study showed that human centered verification enhances the reliability of misinformation detection systems in healthcare domain (Feng et al., 2025; Malek et al., 2026). Domain specific health information enhanced contextual reasoning through structured medical evidence (Feng et al., 2025). Human based verification reduced unsupported inference during automatic claims verification (Malek et al., 2026). Human based verifications enhanced trustworthiness and reliability in sensitive healthcare contexts (Feng et al., 2025; Malek et al., 2026). AI driven support dashboards through the involvement of humans supported in actionable public health intelligence to assist health authorities and policymakers to take evidence based decisions (Hussain et al., 2023). The accuracy of big data and sentiment analytics depends on human supervised decision frameworks apart from algorithmic performance in the current AI dominant age (Feng et al., 2025; Hussain et al., 2023; Malek et al., 2026).

Table 8 illustrates the major and their aligned sub-factors influencing the adoption of big data analytics (BDA) and sentiment analysis (SA) for health misinformation detection.

**Table 8:** Factors influencing the adoption of BDA and SA for health misinformation detection.

Major factor	Sub-factors influencing health misinformation detection
Advanced deep learning architectures	1. Deep neural representation learning techniques
	2. Large language models
	3. Contextual reasoning
	4. Domain specific fine tuning
	5. Machine learning frameworks
	6. Semantic abstraction
	7. Semantic representations
	8. Attention mechanisms
	9. Contextual feature extraction
	10. Multi classification strategies
Feature engineering	1. Authentic data
	2. Data integration
	3. High quality data
	4. Heterogeneous users' interactions
	5. Sentiment score extraction
	6. Feature optimization
	7. Automatic feature selection
	8. Structured and unstructured datasets
	9. Entity recognition
	10. Domain specific health lexicons and semantic normalization
Sentiment intelligence	1. Sentiment analysis
	2. Contextual semantic modeling
	3. Emotion aware features
	4. Topic sensitive misinformation evaluation
	5. Health aware sentiment classification
	6. Linguistic extraction
	7. Semantic extraction
	8. Language based contextual meanings
	9. User behaviors
	10. Domain specific health knowledge
Real-time analytics	1. Cloud based analytics
	2. Large health datasets processing
	3. Real time streaming analytics
	4. Immediate identification of health misinformation
	5. Real time stream processing
	5. Monitoring of fake health information
	6. Cloud based storage
	7. Data visualization
	8. Internet of things (IoT) powered ecosystems
	9. Scalable infrastructure
Human-centered verification	1. Domain specific health information
	2. Contextual reasoning through medical evidence
	3. Structured medical evidence
	4. Human based verification
	5. Automatic claims verification
	6. Trustworthiness
	7. Reliability
	8. AI driven support dashboards
	9. Human supervised decision frameworks
	10. Evidence based decision making

### 3.4 Challenges associated with the implementation of big data and sentiment-driven health misinformation detection systems

The results displayed that annotation limitations, scalability constraints, domain adaptation challenges, ethical concerns, and sustainability challenges negatively impacted the adoption of big data and sentiment driven health misinformation detection systems. These challenges are elaborated as follows:

#### 3.4.1 Annotation limitations

Annotation limitations are a key challenge to detect health misinformation on social media platforms effectively. Data heterogeneity is a key adoption challenge as health misinformation is scattered across various platforms with diverse contextual, structural, linguistic, and behavioral patterns (Ahmed and Ahmed, 2023; Di Sotto and Viviani, 2022). The shortage of high-quality annotated health-related sentiment and misinformation datasets creates barriers for developing and evaluating integrated detection systems (Arias et al., 2022; Feng et al., 2025). Informal content posted on digital media forums related to health created processing complications in non-English settings (Chatta et al., 2026; Hussain et al., 2023). Sampling and algorithmic biases can reduce the representativeness of sentiment estimates and broader misinformation-detection findings derived from diverse social-media datasets (Cano-Marin et al., 2023; Cynthia et al., 2026). Unstructured datasets, and mixed sentiments increased the possibilities of misclassification (Balshetwar et al., 2023; Kabir, 2022). Manual annotation proved difficult to maintain in a rapidly evolving misinformation in healthcare environments (Morita et al., 2023). Annotation inconsistencies caused problems for effectively detecting health misinformation (Ahmed and Ahmed, 2023; Chatta et al., 2026; Di Sotto and Viviani, 2022).

#### 3.4.2 Scalability constraints

The findings of the study showed that scalability constraints proved a major barrier for large scale adoption of advanced health misinformation detection frameworks (Alarabid et al., 2026; Cavus et al., 2025). Large language models required adequate memory capacity, and processing power for sustainable functioning (Babar et al., 2024; Villanueva-Miranda et al., 2025). Real time analytics produced latency limitations that created complications for quick detection and analytical accuracy (Babar et al., 2024; Kansara and Adhvaryu, 2022). The adoption of cloud based systems increased infrastructure costs and dependency on external resources (Alarabid et al., 2026; Cavus et al., 2025). High dimensional big data environments caused difficulties related to storage optimization and effective model training (Kansara and Adhvaryu, 2024). During health emergencies, resource processing requirements caused complications due to the increase of misinformation volumes in healthcare domain (Hussain et al., 2023; Taha, 2025). Scalability limitations occurred due to the collective effects of streaming data velocity, infrastructure finances, need for real time processing, and model complexity (Alarabid et al., 2026; Cavus et al., 2025; Taha, 2025).

#### 3.4.3 Domain adaptation challenges

The findings showed that domain adaptation challenges cause barriers for effectively detecting health misinformation. Contextual interpretation is a key challenge as misinformation includes emotionally implicit language that may not be effectively managed (Kansara and Adhvaryu, 2024; Zamir et al., 2024). Domain adaptation is required not only because sentiment expressions vary across populations and health contexts, but also because the same sentiment can accompany both factual and misleading information. Therefore, contextual and domain-specific representations are necessary to interpret sentiment signals appropriately within misinformation detection systems (Alonso et al., 2021). Diversity in sentiment expressions across health settings reduced the accuracy ratio of models trained on a single misinformation context (Alonso et al., 2021; Arias et al., 2022). Language related processing complexities negatively affected multilingual detection performance (Chatta et al., 2026; Liu et al., 2019). To differentiate misinformation from authentic mental health expressions caused contextual complication due to the overlapping of linguistic and emotional patterns (Arora et al., 2026). The fast nature of the content posted on social media platforms caused barriers to efficiently identify health misinformation patterns (George and Baskar, 2024; Shahzad et al., 2022). To successfully detect health misinformation on the social media, it was essential to effectively adopt context models capable of managing diverse linguistic patterns and domain specific semantic ambiguity (Arora et al., 2026; George and Baskar, 2024; Kansara and Adhvaryu, 2024).

#### 3.4.4 Ethical concerns

Ethical and privacy concerns are key adoption barriers during the analysis of sensitive patient-generated and mental health-related datasets (Arora et al., 2026; Wreyford et al., 2026). Unreliable automated reasoning and unsupported health recommendations caused challenges for detecting misinformation (Alarabid et al., 2026; Malek et al., 2026). The unavailability of standards reduced transparency and created trust issues in automatic misinformation classification related to health contexts (Alarabid et al., 2026; Villanueva-Miranda et al., 2025). Cyber-security issues were linked to the adoption of big data analytics and sentiment analysis for health misinformation detection (Azzaoui et al., 2021; Taha, 2025). Biases in social media datasets created concerns related to ethical AI adoption within public health decision-making (Cano-Marin et al., 2023; Villanueva-Miranda et al., 2025). Inadequate external knowledge sources caused trust issues in AI based health misinformation detection systems (Alarabid et al., 2026; Malek et al., 2026; Villanueva-Miranda et al., 2025).

#### 3.4.5 Sustainability challenges

Sustainability challenges are linked to the adoption of big data analytics and sentiment analysis for health misinformation detection. Operational integration is a key challenge in AI based misinformation monitoring system into existing healthcare systems (Hussain et al., 2023). The lack of coordination among key stakeholders including healthcare decision makers, policy makers, and technical systems create sustainability issues (Hussain et al., 2023). The unavailability of a comprehensive infodemic surveillance frameworks caused barriers for the continuous misinformation monitoring (Hussain et al., 2023). Architectural complications happened due to integration issues of multiple analytics, storage, classification modules, and visualization (Babar et al., 2024; Morita et al., 2023; Sharma et al., 2025). Cross-platform inconsistencies complicated the adoption of unified context-aware detection frameworks (Liu et al., 2019; Shahzad et al., 2022). The findings demonstrated that long-term sustainability depends on institutional governance, interoperable infrastructure, and integrated public health surveillance ecosystems (Hussain et al., 2023; Morita et al., 2023; Sharma et al., 2025). Figure 4 shows the challenges for the adoption of big data analytics (BDA) and sentiment analysis (SA) for detecting misinformation in health domain.

**Figure 4:**
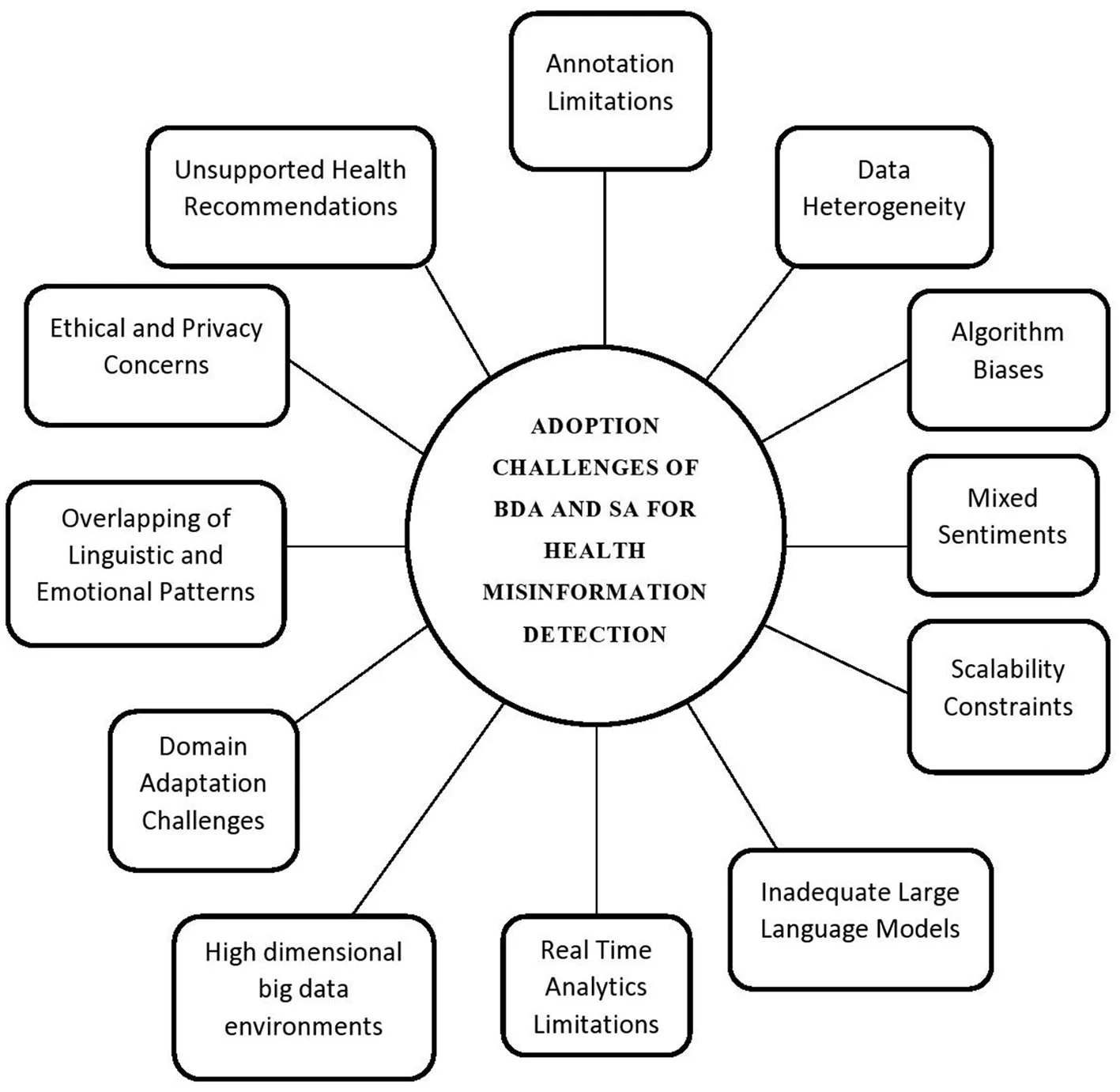
Adoption challenges of BDA and SA for health misinformation detection.

## 4 Discussion

The study aimed to identify the factors influencing big data analytics (BDA) and sentiment analysis (SA)-based approaches for health misinformation detection and the challenges associated with implementing big data and sentiment-supported misinformation detection systems in healthcare information environments. The systematic literature review (SLR) synthesized evidence from 30 peer-reviewed studies and conference proceedings. Rather than treating the identified factors as isolated technical elements, the findings indicate that effective health misinformation detection depends on their interaction within a broader data, analytical, human, and governance ecosystem.

A major finding of the review is the transition from conventional feature-based machine learning toward context-aware deep learning, transformer-based architectures, and large language models. Earlier studies emphasized linguistic, statistical, and behavioral features for misinformation classification (Alonso et al., 2021; Liu et al., 2019), whereas more recent investigations increasingly employ contextual representations generated through BERT, transformer models, and large language models (Alarabid et al., 2026; Arora et al., 2026; Cavus et al., 2025; Malek et al., 2026). This progression suggests that the increasing contextual and linguistic complexity of health misinformation has reduced the sufficiency of conventional lexical approaches. However, the evidence does not imply that more sophisticated models necessarily provide reliable detection in all settings. Their performance remains dependent on representative datasets, contextual adaptation, external evidence, and appropriate validation.

Another important finding concerns the role of sentiment analysis. The reviewed literature indicates a growing convergence between sentiment analysis and broader misinformation analytics. Earlier sentiment-oriented studies tended to treat emotional polarity as a relatively independent analytical variable (Alonso et al., 2021; Marques et al., 2022), whereas more recent approaches integrate sentiment-derived information with semantic representations, contextual embedding, and behavioral indicators (Ahmed and Ahmed, 2023; Cavus et al., 2025; Zamir et al., 2024). This finding requires a critical qualification which indicates that the presence of positive, negative, or neutral sentiment cannot independently establish whether health information is factual or misleading. Sentiment is therefore better interpreted as a complementary signal that may help characterize how information is communicated, received, and disseminated. Factuality assessment requires additional evidence, including contextual semantics, source credibility, external knowledge, temporal propagation, user behavior, and verification mechanisms. This distinction is important because treating sentiment classification as equivalent to misinformation detection could lead to conceptual and methodological overstatement.

The review also indicates that data quality may be as important as, or in some circumstances more consequential than, algorithmic complexity. Although several recent studies report strong predictive performance from deep learning and LLM-based architectures (Alarabid et al., 2026; Cavus et al., 2025; Malek et al., 2026), these approaches depend on representative, balanced, accurately annotated, and domain-specific datasets (Di Sotto and Viviani, 2022; Feng et al., 2025; Sharma et al., 2025). Evidence from Morita et al. (2023) and Hussain et al. (2023) similarly highlights the importance of expert-validated information and appropriate data infrastructures. Thus, the findings challenge a purely technology-centered interpretation of misinformation detection which shows that increasing model sophistication cannot fully compensate for weaknesses in data quality, annotation, contextual representation, or domain adaptation. A further contribution of the review is the identification of health misinformation detection as an increasingly dynamic rather than static classification problem. Earlier approaches generally emphasized classification of already disseminated content (Ahmed and Ahmed, 2023; Liu et al., 2019), whereas more recent research emphasizes streaming analytics, continuous surveillance, dashboard-based monitoring, anomaly detection, and predictive assessment (George and Baskar, 2024; Hussain et al., 2023; Morita et al., 2023). This development is particularly relevant to health emergencies, during which misinformation narratives can change rapidly. Nevertheless, continuous or real-time detection introduces additional requirements for computational capacity, infrastructure, model updating, and monitoring. Real-time capability should therefore be regarded as an operational requirement that must be balanced against scalability and resource constraints rather than as an inherent indicator of system superiority.

The implementation challenges identified in this review demonstrate that multilingual communication, sarcasm, implicit emotions, concept drift, platform-specific language, and contextual variation can reduce the transferability of models across datasets and information environments (Ahmed and Ahmed, 2023; Balshetwar et al., 2023; Chatta et al., 2026; Kansara and Adhvaryu, 2024; Zamir et al., 2024). At the same time, transformer architectures and LLMs may require substantial computational resources, storage capacity, cloud infrastructure, and financial investment (Alarabid et al., 2026; Cavus et al., 2025; Taha, 2025; Villanueva-Miranda et al., 2025). Consequently, there is a potential trade-off between analytical sophistication and practical scalability. This issue is particularly relevant to healthcare environments with limited technological and financial resources. It suggests that future research should examine computationally efficient and context-adaptive approaches rather than focusing exclusively on predictive performance.

The findings of this review are broadly consistent with earlier reviews that have emphasized the importance of data science, machine learning, natural language processing, and contextual analysis in health misinformation detection (Di Sotto and Viviani, 2022; Liu et al., 2019). However, the present review extends this literature in several respects. Firstly, whereas previous reviews have largely concentrated on the technological approaches used to identify misinformation, the current synthesis brings together analytical factors and implementation challenges within the same evidence base. This provides a broader perspective on not only what techniques are being used but also the conditions and barriers affecting their implementation. Secondly, the review provides a more explicit interpretation of the relationship between sentiment analysis and factuality assessment. Existing literature has demonstrated the usefulness of sentiment and emotion-related features in analyzing health communication (Alonso et al., 2021; Shahzad et al., 2022), however, the present synthesis emphasizes that sentiment should not be treated as a direct proxy for truthfulness. This distinction assists in clarifying the conceptual boundary between sentiment classification and misinformation detection and addresses an important methodological issue identified across the reviewed studies. Thirdly, compared with reviews that primarily examine misinformation detection as a classification task, the current review emphasizes the movement toward integrated, adaptive, human-supported, and governed detection ecosystems. The combination of heterogeneous data acquisition, intelligent data management, AI-driven analysis, human validation, and adaptive governance is therefore presented as a conceptual synthesis of the evidence rather than simply another classification architecture. This broader perspective is particularly relevant because the challenges identified in the literature extend beyond model accuracy to include annotation, scalability, domain adaptation, privacy, bias, cyber-security, interoperability, and sustainability.

The comparison with existing reviews should be interpreted cautiously. The relatively small number of included studies, methodological heterogeneity, English-language restriction, and absence of quantitative synthesis limit the extent to which the present review can establish that one analytical approach is superior to another. The contribution of the study is therefore primarily integrative and conceptual, rather than a statistical demonstration of comparative effectiveness. On the whole, the evidence suggests that future health misinformation detection research should move beyond isolated algorithmic classification toward systems that combine contextual intelligence, heterogeneous data, sentiment-derived signals, evidence assessment, human supervision, and adaptive governance. The proposed framework in Figure 5 reveals this synthesis by organizing these requirements into an evidence-informed conceptual structure. Its empirical effectiveness, however, remains to be established through future implementation and validation studies.

**Figure 5:**
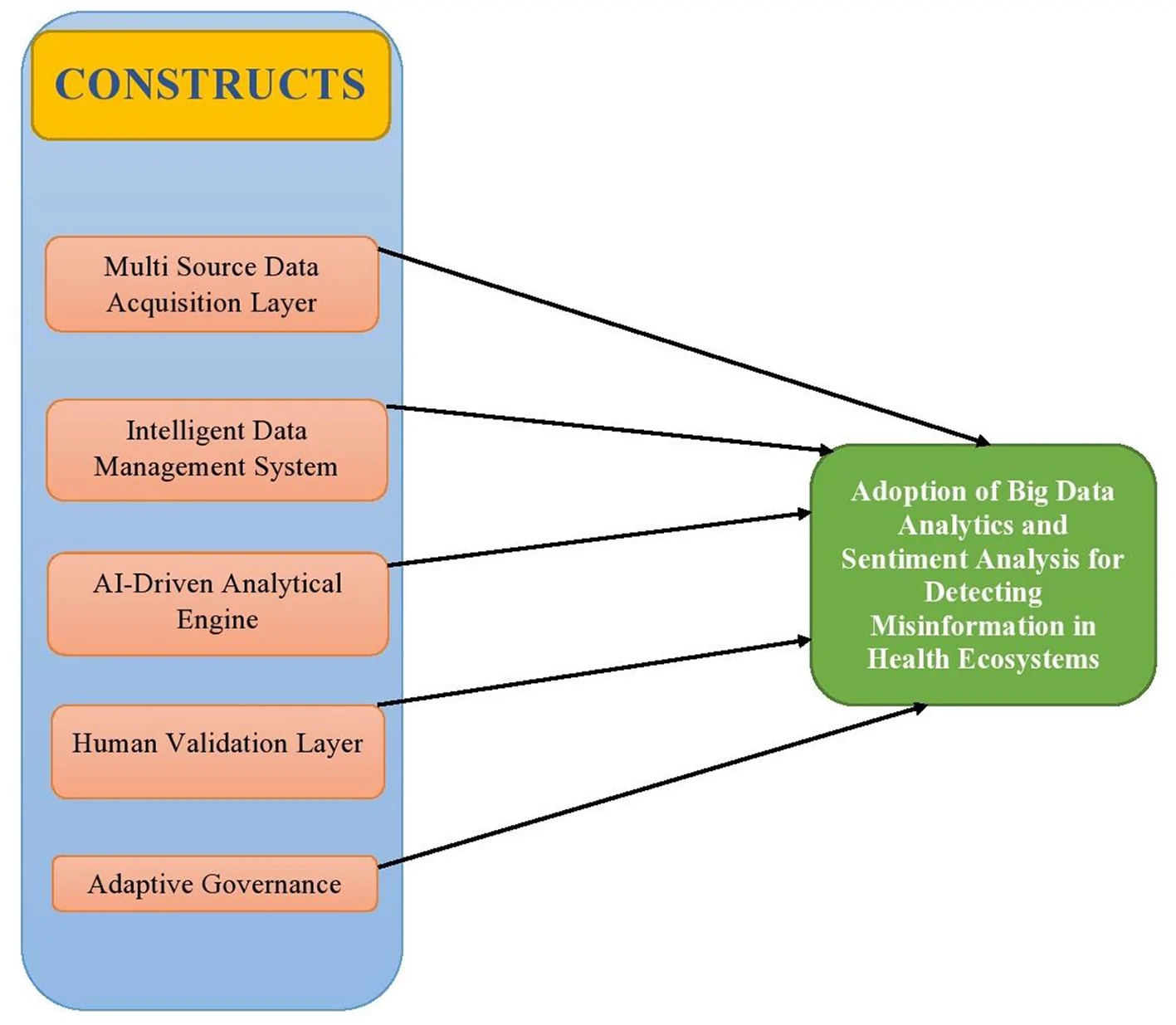
Framework for adopting BDA and SA health misinformation detection.

## 5 Framework for big data analytics and sentiment analysis in health misinformation detection

Based on the synthesis of the reviewed studies, an evidence-informed conceptual framework for big data analytics (BDA) and sentiment analysis (SA) in health misinformation detection is proposed. The framework was developed by synthesizing the recurring factors influencing BDA- and SA-based misinformation detection and the challenges associated with their implementation. Rather than being derived from a single existing theoretical model, the five components were conceptually developed by grouping the reviewed evidence according to the functional requirements of a health misinformation detection ecosystem. These components comprise: (1) multi-source data acquisition layer, (2) intelligent data management system, (3) AI-driven analytical engine, (4) human validation layer, and (5) adaptive governance.

The derivation of the five components follows a sequential and interconnected logic. Evidence concerning heterogeneous information sources, diverse digital platforms, and the need for broader contextual coverage informed the multi-source data acquisition layer. Findings related to data quality, annotation limitations, feature engineering, multilingual processing, semantic representation, and domain adaptation informed the intelligent data management system. Evidence concerning advanced deep learning, machine learning, large language models, sentiment-derived signals, contextual reasoning, and real-time analytics informed the AI-driven analytical engine. Findings emphasizing human supervision, explainability, expert verification, evidence assessment, and trust informed the human validation layer. Finally, evidence concerning ethical and privacy concerns, bias, cyber-security, scalability, interoperability, sustainability, continuous monitoring, and institutional coordination informed the adaptive governance component. Thus, the proposed framework represents an evidence-informed synthesis of the major technological, analytical, human, and governance requirements identified in the reviewed literature.

The framework is proposed as a conceptual structure informed by the reviewed evidence, rather than as an empirically validated model. The reviewed studies provide support for the relevance of individual factors and mechanisms, but the present review does not empirically test the five components as an integrated framework. Therefore, the framework is intended to organize the evidence and provide a coherent conceptual basis for future implementation and empirical investigation. Its effectiveness, predictive capability, and relationships among individual components require validation through future empirical research.

### 5.1 Multi-source data acquisition layer

The multi-source data acquisition layer represents the foundational input component of the framework. Its inclusion was derived from evidence concerning data heterogeneity, diverse information environments, cross-platform variation, and the need for contextual coverage identified across the reviewed studies. Health misinformation is disseminated through multiple digital environments, including social media platforms, online news portals, healthcare repositories, public health databases, IoT-enabled health systems, and patient-generated digital content. The reviewed literature consequently indicates the importance of considering diverse sources when constructing datasets for health misinformation analysis (Arias et al., 2022; Azzaoui et al., 2021; Feng et al., 2025; Morita et al., 2023).

This component also responds to the annotation limitations and data heterogeneity challenges identified in the review. Health-related content varies considerably in structure, language, context, and user behavior across platforms. The scarcity of high-quality annotated datasets, multilingual content, informal health communication, sampling limitations, and annotation inconsistencies can constrain the representativeness and reliability of subsequent analysis (Ahmed and Ahmed, 2023; Chatta et al., 2026; Di Sotto and Viviani, 2022; Feng et al., 2025). Accordingly, the framework positions multi-source acquisition as the initial stage through which heterogeneous health information can be systematically collected for subsequent processing and analysis. The role of this layer is therefore not to establish misinformation independently, but to provide sufficiently diverse and contextually relevant information for downstream analytical processes. Its inclusion directly reflects the reviewed evidence that fragmented and heterogeneous health information environments constitute an important consideration in developing BDA and SA-based misinformation detection systems.

### 5.2 Intelligent data management system

The intelligent data management system was derived from recurring evidence concerning data quality, annotation limitations, feature engineering, multilingual processing, entity recognition, semantic representation, and domain adaptation. The reviewed studies demonstrate that the quality, structure, and contextual representation of data are important considerations for downstream misinformation analysis (Chatta et al., 2026; Di Sotto and Viviani, 2022; Sharma et al., 2025; Zamir et al., 2024). This component incorporates data cleaning, normalization, multilingual processing, feature engineering, entity recognition, semantic enrichment, and contextual preprocessing. These functions respond directly to the challenges identified in the review, including heterogeneous and unstructured datasets, annotation inconsistencies, mixed sentiments, multilingual content, and domain-specific semantic ambiguity (Ahmed and Ahmed, 2023; Balshetwar et al., 2023; Chatta et al., 2026; Di Sotto and Viviani, 2022).

Feature engineering is particularly relevant because the reviewed studies identified sentiment scores, entity-related information, health-specific lexicons, semantic representations, and other extracted characteristics as useful analytical features (Arias et al., 2022; Babar et al., 2024; Shahzad et al., 2022). At the same time, the literature indicates that sentiment-related characteristics cannot be interpreted independently of context. The same emotional expression may occur in both factual and misleading health information. Consequently, this component is designed conceptually to preserve and integrate contextual, linguistic, semantic, and domain-specific information rather than reducing health content to isolated sentiment categories. The intelligent data management system therefore provides the conceptual bridge between heterogeneous data acquisition and subsequent AI-based analysis. Its inclusion displays the reviewed evidence that reliable analytical outcomes depend on the sophistication of algorithms and on the quality, representation, and contextual preparation of the underlying data.

### 5.3 AI-driven analytical engine

The AI-driven analytical engine was derived from the strongest cluster of evidence concerning advanced deep learning architectures, machine learning, transformer models, large language models, semantic representations, sentiment-derived signals, contextual reasoning, and real-time analytics. The reviewed studies report the use of increasingly advanced computational approaches for analyzing and classifying health misinformation (Alarabid et al., 2026; Arora et al., 2026; Babar et al., 2024; Cavus et al., 2025; Villanueva-Miranda et al., 2025). The analytical engine conceptually integrates machine learning, deep learning, large language models, transformer architectures, sentiment analysis, knowledge graphs, topic modeling, graph-based analytics, and contextual reasoning. The purpose of bringing these approaches is to reveal the multidimensional nature of health misinformation identified in the reviewed literature, where semantic, linguistic, emotional, behavioral, temporal, source-related, and contextual characteristics may all provide relevant analytical information. Within this component, sentiment analysis is explicitly positioned as an auxiliary analytical signal rather than a standalone misinformation detection mechanism. Sentiment-derived information can capture emotional polarity, attitudes, opinions, and affective characteristics of health-related content and may complement semantic and contextual representations (Alonso et al., 2021; Shahzad et al., 2022). However, sentiment polarity alone cannot establish whether a health claim is factual or misleading because misinformation may be expressed using positive, negative, or neutral sentiment. Factuality assessment consequently requires additional contextual reasoning, source assessment, evidence retrieval, domain-specific knowledge, and/or human verification (Liu et al., 2019; Malek et al., 2026).

Real-time analytics are also incorporated into this component because the reviewed studies identify the velocity and continuous emergence of online health information as important considerations for misinformation monitoring (Babar et al., 2024; Kansara and Adhvaryu, 2024; Taha, 2025). However, the review also identified scalability constraints, computational requirements, processing latency, storage demands, and infrastructure costs (Alarabid et al., 2026; Cavus et al., 2025). Therefore, the framework does not assume that real-time or advanced AI analytics can be implemented without adequate technical and organizational resources. The AI-driven analytical engine consequently represents the computational core of the proposed framework, while its outputs remain subject to contextual interpretation and validation through the subsequent human validation layer.

### 5.4 Human validation layer

The human validation layer was derived from evidence concerning human-centered verification, explainability, expert oversight, evidence assessment, trust, and limitations of automated reasoning. The reviewed studies indicate that automated systems may generate unsupported inferences and unreliable outputs, particularly in sensitive healthcare contexts where incorrect information classification can have serious implications (Feng et al., 2025; Malek et al., 2026). This component incorporates explainable AI, confidence estimation, evidence retrieval, expert verification, retrieval-augmented evidence generation, and human-in-the-loop decision support. These elements were derived from findings emphasizing the importance of human involvement and domain-specific health knowledge in interpreting automated analytical outputs (Feng et al., 2025; Hussain et al., 2023; Malek et al., 2026).

The human validation layer is therefore conceptually positioned as a mechanism through which analytical results can be reviewed, interpreted, and contextualized before they inform sensitive health-related decisions. Human involvement is particularly important where automated systems encounter ambiguous language, conflicting evidence, culturally diverse expressions, or insufficient external knowledge. This layer consequently addresses the ethical and practical limitations of relying exclusively on automated classification. Rather than assuming that human validation automatically guarantees accuracy, the framework recognizes it as an evidence-informed safeguard that may contribute to more transparent and contextually informed decision-making. The actual contribution of this layer to detection accuracy, trust, or decision quality requires empirical assessment in future research.

### 5.5 Adaptive governance

The adaptive governance component was derived from evidence concerning ethical and privacy concerns, algorithmic bias, cyber-security, scalability, interoperability, sustainability, continuous monitoring, and institutional coordination. These challenges extend beyond the technical performance of individual analytical models and concern the responsible and sustainable operation of health misinformation detection systems. The framework incorporates ethical governance, privacy protection, bias mitigation, regulatory compliance, performance monitoring, model updating, stakeholder coordination, interoperability, and continuous learning. The inclusion of these elements reflects findings that health misinformation detection operates within rapidly changing technological, social, institutional, and regulatory environments (George and Baskar, 2024; Hussain et al., 2023; Morita et al., 2023; Taha, 2025; Villanueva-Miranda et al., 2025; Wreyford et al., 2026).

Adaptive governance responds directly to the sustainability challenges identified in the review. Operational integration among analytical, storage, classification, visualization, and surveillance systems can be complex, while limited coordination among healthcare professionals, policymakers, technical experts, and other stakeholders can constrain long-term implementation (Hussain et al., 2023; Morita et al., 2023; Sharma et al., 2025). Cross-platform inconsistencies and continuously changing misinformation patterns further indicate the need for systems capable of continued monitoring and adaptation. Governance is conceptualized as a cross-cutting component rather than a final stage of the detection process. Ethical, privacy, security, bias, and compliance considerations apply across data acquisition, data management, analytical processing, evidence assessment, human validation, and decision-making.

### 5.6 Integrated conceptual logical of the framework

The five components collectively provide an integrated conceptual architecture derived from the evidence synthesized in this review. The multi-source data acquisition layer addresses the diversity and heterogeneity of health information. The intelligent data management system addresses data quality, annotation, feature engineering, multilingual processing, and contextual representation. The AI-driven analytical engine addresses computational detection through advanced analytical and sentiment-derived techniques. The human validation layer addresses explainability, evidence assessment, expert oversight, and contextual verification. Adaptive governance addresses ethical, organizational, infrastructural, sustainability, and continuous adaptation requirements.

The components should not be interpreted as isolated linear stages. They are conceptually interdependent. Data quality and contextual representation influence analytical processing. Analytical outputs may require human interpretation. Human validation can generate feedback for system improvement; and governance considerations apply across the entire ecosystem. This integrated structure reflects the multidimensional nature of health misinformation identified across the reviewed studies. The proposed framework is evidence-informed and conceptual, and not an empirically validated effectiveness model. The systematic review provides the evidential basis for organizing the identified factors and challenges into the five components. However, it does not test whether their integration produces superior misinformation detection outcomes. Consequently, no causal and effectiveness claims are made regarding the framework as a whole. Future empirical research should evaluate the framework through prototype implementation, expert assessment, comparative system testing, and field-based studies across different healthcare and digital-information environments. Such validation could examine detection accuracy, contextual reliability, explainability, timeliness, stakeholder trust, scalability, and sustainability.

Figure 5 illustrates the evidence-informed conceptual framework for the adoption and implementation of big data analytics (BDA) and sentiment analysis (SA) in health misinformation detection.

## 6 Implications

The study has theoretical, managerial, methodological, and social implications. Theoretically, it contributes an integrated perspective by bringing together technological, data-related, human, and governance considerations in health misinformation detection. Rather than treating sentiment analysis as an independent mechanism for determining misinformation, the study positions sentiment-derived information as a complementary signal within a broader evidence-based analytical process. For management and healthcare decision-makers, the findings emphasize the importance of investing not only in advanced analytical technologies but also in high-quality datasets, scalable infrastructure, human expertise, evidence verification, and responsible AI governance. Methodologically, the study provides an evidence-informed conceptual framework that can guide future empirical investigations of BDA and SA-supported health misinformation detection. Socially, improved integration of contextual analysis, evidence assessment, and human validation may contribute to more responsible identification and communication of potentially misleading health information. However, these implications should be considered conceptual as the proposed framework has not yet been empirically validated.

## 7 Limitations and future research directions

Following are limitations and future research directions of the study:

1. The study was conducted through a systematic literature review (SLR) methodology. Future studies can be conducted through the employment of empirical methods i.e., qualitative, quantitative, and mixed methods designs etc.
2. Thirty peer-reviewed articles and conference proceedings were selected to conduct the SLR on the topic. Books, book chapters, dissertations and other sources of knowledge were not consulted. Future investigators can consider the inclusion of these sources for increasing sample size of the studies.
3. The required studies were selected through digital databases including Web of Science, Scopus, and PubMed. Other sources of knowledge i.e., educational websites, search engines, and institutional repositories were not explored. Future studies can be proceeded using these platforms for offering a broader outlook to readers.
4. In future, a similar study can be conducting by applying bibliometric, content analysis, scoping review, and meta-analysis methods for further contributing methodologically.
5. The proposed framework through Figure 5 can be empirically tested by adopting different research studies including research articles and dissertations.
6. The reviewed literature frequently combines sentiment analysis with broader machine learning, natural language processing, and misinformation detection approaches, which may create conceptual overlap between sentiment classification and misinformation classification. Although the current SLR distinguishes sentiment-derived signals from factuality assessment, the heterogeneity of the included studies may limit direct comparison of the specific contribution of sentiment analysis to misinformation detection. Future empirical studies should separately evaluate sentiment features and other evidence-based features to determine their incremental contribution to misinformation detection performance.
7. The review was restricted to English-language publications, which may have resulted in the exclusion of relevant studies published in other languages. This limitation may be particularly important for health misinformation research because misinformation patterns, sentiment expressions, and detection practices can vary across linguistic and cultural contexts. Future reviews should consider including non-English literature to provide a more comprehensive and internationally representative evidence base.
8. The possibility of publication and database bias cannot be completely excluded. The review primarily relied on studies indexed in Web of Science, Scopus, and PubMed, and therefore relevant evidence published in other databases, repositories, and less visible publication outlets may not have been captured. Furthermore, studies reporting positive or significant findings may be more likely to be published than studies reporting limited or non-significant findings. Future investigations should broaden database coverage and, where appropriate, incorporate additional sources of scholarly and gray literature to reduce potential retrieval and publication bias.
9. The included studies demonstrated considerable methodological and contextual heterogeneity in terms of research designs, datasets, healthcare contexts, digital platforms, analytical techniques, and applications of machine learning, natural language processing, big data analytics, and sentiment analysis. Such heterogeneity limits direct comparison of findings across studies. In particular, sentiment analysis was frequently integrated with broader analytical approaches. Future empirical studies should separately examine sentiment-derived features alongside semantic, behavioral, temporal, source-related, and evidence-based features to determine their specific and incremental contributions.
10. The relatively small evidence base of 30 included studies represents another limitation of the present review. Although the included studies provided sufficient evidence to identify recurring factors, challenges, and conceptual relationships, the relatively limited number of studies may constrain the generalizability and comprehensiveness of the findings. Moreover, the heterogeneity of the available evidence did not facilitate a meaningful quantitative synthesis or meta-analysis. Consequently, the present findings were synthesized narratively and conceptually. Future research should consider quantitative synthesis when a sufficiently large and methodologically comparable body of empirical evidence becomes available.

## 8 Conclusion

This systematic literature review indicates that effective health misinformation detection requires more than increasingly sophisticated analytical algorithms. The evidence suggests that reliable detection depends on the interaction of high-quality and contextually representative data, advanced analytical techniques, sentiment-derived signals, real-time capabilities, human verification, and responsible governance. In particular, sentiment analysis should be understood as a complementary analytical signal rather than a standalone mechanism for determining whether health information is factual or misleading. The review also demonstrates that implementation remains constrained by annotation limitations, scalability, domain adaptation, ethical concerns, and sustainability challenges. These findings indicate that technological sophistication must be accompanied by appropriate data governance, contextual adaptation, human oversight, and organizational capacity. On this basis, an evidence-informed conceptual framework (Figure 5) was developed comprising multi-source data acquisition, intelligent data management, an AI-driven analytical engine, human validation, and adaptive governance. The principal contribution of the study is therefore an integrated conceptual perspective that connects the technological capabilities and implementation requirements of BDA- and SA-supported health misinformation detection. However, because the framework has not been empirically tested and the evidence base is relatively heterogeneous and limited, its effectiveness cannot yet be established. Future empirical research should validate the framework across diverse healthcare and digital-information environments and examine its accuracy, explainability, scalability, adaptability, and practical utility.

## Data Availability

The raw data supporting the conclusions of this article will be made available by the authors, without undue reservation.
